# Tumor Microenvironment in Ovarian Cancer: Function and Therapeutic Strategy

**DOI:** 10.3389/fcell.2020.00758

**Published:** 2020-08-11

**Authors:** Yanfei Yang, Yang Yang, Jing Yang, Xia Zhao, Xiawei Wei

**Affiliations:** ^1^Department of Gynecology and Obstetrics, Key Laboratory of Birth Defects and Related Diseases of Women and Children, Ministry of Education, West China Second Hospital, Sichuan University, Chengdu, China; ^2^Laboratory of Aging Research and Cancer Drug Target, State Key Laboratory of Biotherapy and Cancer Center, National Clinical Research Center for Geriatrics, West China Hospital, Sichuan University, Chengdu, China

**Keywords:** ovarian cancer, tumor microenvironment, anti-angiogenesis therapy, tumor-associated macrophage-targeted strategies, immune checkpoint inhibitors

## Abstract

Ovarian cancer is one of the leading causes of death in patients with gynecological malignancy. Despite optimal cytoreductive surgery and platinum-based chemotherapy, ovarian cancer disseminates and relapses frequently, with poor prognosis. Hence, it is urgent to find new targeted therapies for ovarian cancer. Recently, the tumor microenvironment has been reported to play a vital role in the tumorigenesis of ovarian cancer, especially with discoveries from genome-, transcriptome- and proteome-wide studies; thus tumor microenvironment may present potential therapeutic target for ovarian cancer. Here, we review the interactions between the tumor microenvironment and ovarian cancer and various therapies targeting the tumor environment.

## Highlights

– The tumor microenvironment plays important roles in the progression of ovarian cancer.
– Current “-omic” technology is revealing the molecular landscape of ovarian cancer tumor microenvironment, facilitating future therapeutic strategy.
– Ovarian cancer therapies targeting tumor microenvironment is rapidly developing, targets mainly focusing on cancer-associated fibroblasts, tumor-associated macrophages, angiogenesis and immune checkpoint blockade.

## Introduction

Ovarian cancer is one of the leading causes of common, lethal gynecologic malignancy (Cortez et al., 2018). In 2018 worldwide, there were an estimated 295,414 cases and 184,799 deaths from ovarian cancer (Bray et al., 2018). Because of the lack of an early diagnosis method and the absence of specific early warning symptoms, patients with ovarian cancer are usually diagnosed at an advanced stage and have a poor prognosis (Scarlett and Conejo-Garcia, 2012).

Based on histological origin, ovarian tumors can be categorized into epithelial, germ cell, sex cord, or stromal tumors (Jayson et al., 2014). Around 90% of primary ovarian tumors are of epithelial origin (Colombo et al., 2010; Ledermann et al., 2013), so we mainly focus on evidence of epithelial ovarian cancer in this review. The World Health Organization (WHO) classified epithelial ovarian cancer (EOC) into the following types: serous, mucinous, endometrioid, clear cell, transitional cell, mixed epithelial, undifferentiated, and unclassified (Ledermann et al., 2013). According to architectural features, EOC is also classified into 3 grades by the International Federation of Gynecology and Obstetrics (FIGO) system (Colombo et al., 2010); in serous EOC, FIGO grade 1 is defined as low-grade while FIGO grade 2 and 3 are combined as high-grade (Bodurka et al., 2012). The classification with histosubtypes and grades are with prognostic significance (Ledermann et al., 2013).

The current standardized treatment for ovarian cancer is optimal cytoreductive surgery plus platinum-based chemotherapy with the carboplatin-paclitaxel regimen (Bolton et al., 2012). However, with the development of chemotherapy-resistant and refractory diseases, the sensitivity of chemotherapy has decreased (Lim and Ledger, 2016). Therefore, the long-term survival rate for ovarian cancer has decreased, and the recurrence rate has increased (Lim and Ledger, 2016). Hennessy et al. (2009) reported that despite benefiting from first-line therapy, 75% of patients with advanced ovarian cancer (stage III or IV) have tumor relapse at a median of 15 months from diagnosis. Moreover, for patients with early-stage disease (stage I or II), the long-term survival rates (>10 years) are 80–95%. In contrast, patients with advanced disease (stage III or IV) had a 10–30% long-term survival rate. Therefore, there is an urgent need to find new targeted therapies to improve the treatment efficacy of ovarian cancer. In recent years, the tumor microenvironment (TME) has been reported to play a vital role in the tumorigenesis of ovarian cancer and is considered a possible therapeutic target for ovarian cancer. Here, we review the interactions between the TME and ovarian cancer and various therapies targeting the tumor environment.

## TME in Ovarian Cancer

**FIGURE 1:**
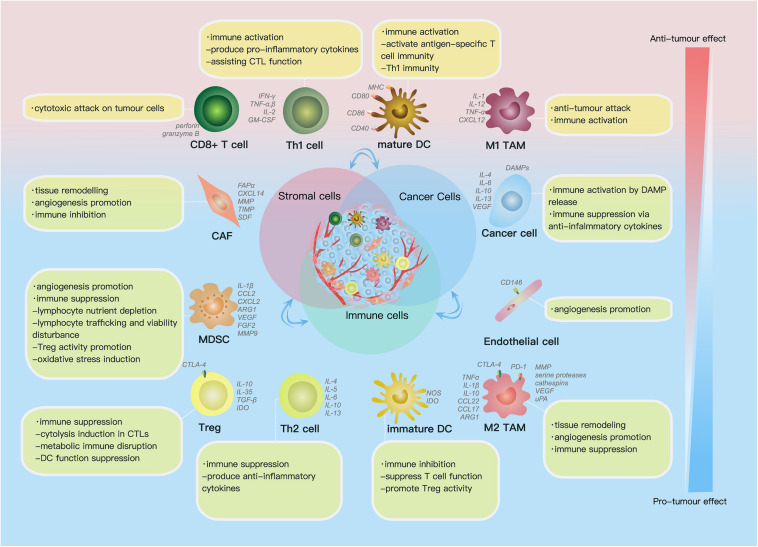
Cell components and functions in the tumor microenvironment (TME). Cell components in the TME can be classified into cancer cells, immune cells and stromal cells; these cells actively interact with each other by molecules they secrete [including cytokines, chemokines, damage-associated molecular patterns (DAMPs) etc.] and receptors they express, such as histocompatibility complex class (MHC) molecules, programmed cell death protein 1 (PD-1), etc., forming an evolving microenvironment. On the continuous spectrum from anti-tumor to pro-tumor effect, different cell components can locate at distinct positions, and the same group of cells may also be re-polarized depending on signals in the TME. The progression or regression of a single tumor site depends on the overall effect of the complex cellular and molecular regulating network in the TME.

The TME comprises (1) the extracellular matrix (ECM), which consists of chemokines, inflammatory cytokines, integrins, matrix metalloproteinases (MMPs) and other secreted molecules, and (2) stromal cells, including cancer cells, cancer stem cells, pericytes, cancer-associated fibroblasts (CAFs), endothelial cells (ECs) and immune cells (Figure 1) (Hanahan and Weinberg, 2011). In this part, we reviewed current findings on the impact of some key components above on ovarian cancer progression.

### Cancer-Associated Fibroblasts

Fibroblasts, which differentiate from mesenchymal-derived cells, are part of the TME (Ishii et al., 2016). They produce various MMPs, tissue inhibitor of metalloproteinases (TIMPs) and most of the proteins comprising the ECM, such as collagens, fibronectin and laminin (Kalluri and Zeisberg, 2006; Erdogan and Webb, 2017). These fibroblasts in the tumor milieu are also called “CAFs.” Additionally, CAFs can transdifferentiate from other cells, such as pericytes, epithelial cells and ECs, via exposure to platelet-derived growth factor (PDGF), tumor-derived transforming growth factor-β (TGF-β), vascular endothelial growth factor (VEGF), basic fibroblast growth factor (bFGF), MMPs and reactive oxygen species (ROS) (Cai et al., 2012; Yu Y. et al., 2014; Denton et al., 2018).

CAFs are known to promote tumor progression via various mechanisms. CAFs can enhance tumor cell proliferation, invasion and migration. Sjoberg et al. (2016) showed that CAFs highly expressed CXCL14, which was an important factor in promoting cancer growth. CAFs also express the fibroblast activation protein α (FAP). Yang’s study indicated that FAPα enhanced the migration and invasion ability of HO-8910PM cells (a highly metastatic ovarian cancer cell line) Additionally, FAPα increased HO-8910PM cell proliferation.

CAFs promote immune inhibition and angiogenesis. Givel et al. (2018) found that CAFs increase the infiltration of FOXP3+ regulatory T lymphocytes (Tregs) at the tumor site, which exerts immune suppression effect in the tumor milieu. Additionally, in Orimo’s research, CAFs have high expression of stromal cell-derived factor-1 (SDF-1). Released SDF-1 promotes angiogenesis and tumor proliferation in a paracrine fashion (Orimo et al., 2005).

CAFs also increase platinum resistance and accelerate recurrence. Fauceglia’s study showed that CAFs expressed the FAP α. By analyzing 338 EOC tissues, they found that the overexpression of FAP acted as a hallmark for platinum resistance. Additionally, patients with FAP+ stroma had a shortened recurrence compared to that of patients with FAP- stroma (Mhawech-Fauceglia et al., 2015).

Several studies have indicated that CAFs was a biomarker of poor prognosis in ovarian cancer (Yang et al., 2013; Mhawech-Fauceglia et al., 2015; Zhao et al., 2017; Givel et al., 2018). In Givel’s study of CAFs in high-grade serous ovarian cancers (HGSOC), the results showed that the expression of CXCL12β and the infiltration of CAF-S1 (a subtype of CAFs) implied a dismal prognosis (Givel et al., 2018). Despite that accumulating studies have demonstrated the pro-tumor progression impact of CAFs, it is worthy of notifying that there are different subtypes of CAFs with heterogenous function status. Recently, Hussain et al. (2020) found 2 CAF subsets distinguished by the FAP expression level. The FAP-high CAF subtype, instead of the FAP-low subtype, was found to aggressively enhance tumor progression and negatively influence patient outcomes, which shed light on therapeutic strategies involving CAF modulation to consider CAF status in patient selection.

CAFs are a crucial cell population in the tumor microenvironment. CAFs promote the proliferation, invasion and migration of cancer cells and stimulate angiogenesis by coordinating with other cells. A deeper understanding of CAFs is needed to better understand how CAFs affect the tumor microenvironment.

### Endothelial Cells

Endothelial cells (ECs) are components of the TME. Lining the vessels, ECs are crucial for transporting oxygen and nutrients and are closely associated with angiogenesis (Carmeliet and Jain, 2000; Hanahan and Weinberg, 2011). As we all know, angiogenesis is a complicated process accommodated by angiogenesis activators and inhibitors. Angiogenesis activators include VEGF, FGF-2, PDGF, TGFα and TGFβ, TNF-α, prostaglandin E2 and Interleukin 8 (IL-8). The angiogenesis inhibitors contain angiopoietin (Angs), Thrombospondin 1 (TSP-1) and endostatin. Moreover, the signal-transducing network of endothelial cells is associated with VEGF, FGF and Angs signals (Cross and Claesson-Welsh, 2001; Ahmed and Bicknell, 2009).

VEGF is a protein family consisting of VEGF-A, VEGF-B, VEGF-C, VEGF-D, VEGF-E and PLGF (placental growth factor). It is regulated by the ischaemia/hypoxia-induced genes (HIFs), epidermal growth factor (EGF) and PDGF (Semenza, 2000). There are three receptors for VEGF: VEGF receptors 1 (VEGFR1), VEGFR2 and VEGFR3. VEGFR1 and VEGFR2 are mainly expressed on ECs and are receptors for VEGF-A (Hagberg et al., 2010). Additionally, VEGFR1 is also a receptor for VEGF-B and PLGF. VEGFR3 is a receptor for VEGF-C and VEGF-D. Neuropilins (NRP1 and NRP2) are coreceptors for the VEGF family. With the help of NRP1, the binding affinity between VEGF-A and PLGF and VEGFR2 increases (Ferrara and Adamis, 2016). Similarly, with the effect of NRP2, VEGF-C, and VEGF-D have increased binding affinity with VEGFR-3. It is well known that the VEGF family is implicated in the adjustment of angiogenesis and lymphangiogenesis (Tammela and Alitalo, 2010; Apte et al., 2019). Among them, VEGF-A is crucial for angiogenesis, while VEGF-C and VEGF-D regulate lymphangiogenesis (Tammela and Alitalo, 2010; Apte et al., 2019).

Angs also a protein family consisting of Ang-1, Ang-2, Ang-3, and Ang-4. Through combined with the receptors of Angs “TIEs,” they perform different functions in angiogenesis. Ang-1 and -4 can bind to TIE2 and stimulate the tyrosine phosphorylation of TIE2. On the contrary, Ang-2 and -3 can competitive combined TIE2 without stimulating tyrosine phosphorylation, which stopping the signal transduction of angiogenesis (Sallinen et al., 2010; Lin et al., 2011; Sallinen et al., 2014). However, other study indicated that with Ang-1, Ang-2 block the TIE2 signaling, while Ang-2 induce the TIE2 signaling without Ang-1 (Yuan et al., 2009). Additionally, Ang-1 promotes the maturation and stabilization of vessel, while Ang-2 destabilize the stabilized vessel (Tse et al., 2003).

VEGF indicate poor clinical outcomes (Wimberger et al., 2014; Shen et al., 2017; Sopo et al., 2019). Wimberger et al. found that by Kaplan-Meier analyses, VEGFR1 expression was closely related to decreased overall survival (OS) and progression-free survival (PFS) (Wimberger et al., 2014). Sopo’ study discovered that VEGFR1, VEGF-A and VEGF-D were highly expressed in omental metastases compared to expression in primary ovarian epithelial tumors. Interestingly, patients with low VEGF-A expression were more likely to have a poor prognosis. Patients with high VEGF-C expression were related to a short PFS (Sopo et al., 2019).

The CD146 expression in the membrane of ECs promotes the migration of ECs and angiogenesis. CD146 is an endothelial biomarker and the extracellular domain of CD146 directly interacts with VEGFR2. Yan’s study demonstrated that CD146 can promote angiogenesis (Yan et al., 2003). Subsequently, Jiang’s report indicates CD146 promotes the migration of ECs and the formation of microvasculature by enhancing VEGFR2 phosphorylation and downstream signaling (AKT/p38 MAPKs/NF-κB) activation (Jiang et al., 2012). Interestingly, Zhou’s research indicated that the gene and protein levels of CD146 and VEGFRA were increased in patients with EOC compared to those of non-cancer patients (Zhou et al., 2019).

Enhanced Angs expression increase relapse and decrease survival time (Sallinen et al., 2010, 2014; Lin et al., 2011). Sallinen et al. observed that patients with ovarian carcinoma had higher Ang2 levels compared to those of patients with benign ovarian tumors. Furthermore, by analyzing the Kaplan–Meier curves, they found that increased Ang-2 levels (>2.7 ng/ml) were a biomarker for poor recurrence-free survival (Sallinen et al., 2010). Subsequently, they discovered that the expression levels of Ang-1 and Ang-2 were 26 and 44%, respectively, higher in women with ovarian cancer than in normal women. Increased Ang-2 expression was significantly related to advanced stage and grade of cancer and relapse of ovarian cancer. Additionally, elevated Ang-2 expression is a predictor of poor OS and short PFS (Sallinen et al., 2014).

As an important part of the tumor microenvironment, ECs are closely related to angiogenesis. VEGF and Angs are crucial regulators in angiogenesis. Both VEGF and Angs are associated with poor clinical outcomes, which provide possible targets for treatment.

### Immune Cells

Immune cells include macrophages, dendritic cells (DCs), neutrophils, mast cells, myeloid-derived suppressor cells (MDSCs) and lymphocytes (Hanahan and Weinberg, 2011). They play significant roles both in tumor progression and tumor suppression, participating in evolving processes of tumorigenesis, metastasis, and angiogenesis by producing various signaling molecules, such as EGF, VEGF, MMP-9, IFNs, ILs, etc.

#### Macrophages

Macrophages are an essential population of immune cells that participate in inflammation and tumourigenesis (Grivennikov et al., 2010). Among them, macrophages residing in tumors are termed as tumor-associated macrophages (TAMs). TAMs can derive from resident macrophages or infiltrating macrophages from bone marrow monocytes circulating in the blood (Ghosn et al., 2010).

Depending on stimuli in the TME, TAMs can present two main phenotypes: the anti-tumor M1 macrophages and pro-tumor M2 macrophages (Sica et al., 2008; Grivennikov et al., 2010; Qian and Pollard, 2010; Sica, 2010; Gupta et al., 2018). When stimulated with interferon-gamma (IFN-γ), bacterial lipopolysaccharide (LPS) and granulocyte-macrophage-colony-stimulating factor (GM-CSF), monocytes differentiated into M1 macrophages, which can secrete IL-1, IL-12, TNFα and CXCL12 (Sica et al., 2008; Ramanathan and Jagannathan, 2014). M1 macrophages possess cytotoxicity, tumor suppression and immune-stimulation functions (Galdiero et al., 2013).

When stimulated with cytokines, including IL-4, IL-10, and IL-13, monocytes differentiated into M2 macrophages (Leyva-Illades et al., 2012; van Dalen et al., 2018). In the immune escape stage, the tumor macroenvironment maintains immunosuppression due to the secretion of many growth factors and cytokines, such as IL-4 and IL-13, by cancer cells. The immunosuppressive state accelerates monocytes to M2 macrophages; M2 macrophages, in turn, can promote tumor growth (Gordon, 2003; Roy and Li, 2016).

In ovarian cancer, TAMs are predominantly M2 macrophages, associating with tumor invasion, angiogenesis, metastatic disease and early recurrence (Pollard, 2004; Reinartz et al., 2014; Yin et al., 2016). They produce and secrete cytokines, which have immunosuppressive effects, such as IL-1R decoy, IL-10, CCL17 and CCL22 (Gordon, 2003). Via several mechanisms, they suppress adaptive immunity (Li et al., 2007; Noy and Pollard, 2014). Firstly, M2 macrophages can inhibit the proliferation of T cells and accelerate the immunosuppression of Treg cell transport to tumors by producing the chemokine CCL22 (Li et al., 2007). Secondly, M2 macrophages express the ligand receptors for CTLA-4 and PD-1. The activation of PD-1 and CTLA-4 inhibits cytotoxic function and regulates the cell cycle of T cells (Noy and Pollard, 2014). Then, M2 macrophages can also inhibit the activation of T cells through the depletion of L-arginine, which plays an essential role in T cell function (Galdiero et al., 2013). Arginase I (ARG1), a hallmark of M2 macrophages, is an L-arginine processing enzyme. In the TME, ARG1 decomposes L-arginine into L-ornithine and urea. The depletion of L-arginine suppresses the re-expression of the CD3 ζ chain, which is internalized by antigen stimulation and T cell receptor (TCR) signaling (Rodriguez et al., 2004).

Aside from immune suppression, M2 macrophages also take part in tissue repair, ECM remodeling and angiogenesis, which are processes involved in tumor progression as well (Mantovani et al., 2002; Coffelt et al., 2010; Ruffell et al., 2012; Finkernagel et al., 2016; Roy and Li, 2016). They can restructure ECM and regulate ECM components by degrading ECM via producing MMPs, serine proteases and cathepsins (Ruffell et al., 2012), which may facilitate tumor cell migration, invasion and metastasis. Additionally, they can secrete VEGF-A, which is an angiogenic factor, and produce proangiogenic cytokines, such as IL-1β, TNFα and uPA (urokinase-type plasminogen activator) (Roy and Li, 2016). In M2 macrophages, there is a subtype expressing TIE2, a tyrosine kinase receptor. The TIE2 macrophages are involved in angiogenesis (Fagiani and Christofori, 2013). These TIE2 macrophages recruited by CCL3, CCL5, CCL8, and TIE2-ligand Ang 2 are considered the most important reason for tumor vascularization because the deficiency of this cell type restricts the angiogenic switch (Ngambenjawong et al., 2017).

TAMs are plastic. The simple dichotomy of M1/M2 macrophages cannot account for the complexity of TAM heterogeneity (Ostuni et al., 2015). Transcriptome analysis uncovered a spectrum model of TAMs (Xue et al., 2014). M1 and M2 macrophages can be regarded as two ends of a continuum with wide ranges of functional states (Mantovani et al., 2002; Ostuni et al., 2015); the sub-populations of TAMs in between the two ends can share features of both M1 and M2 types (Qian and Pollard, 2010). For example, recently Singhal et al. (2019) found that TAMs could co-express M1/M2 markers, together with T cell co-inhibitory and co-stimulatory receptors.

The dynamic nature of the TME cellular environment gives a basis for the plasticity of TAMs. Macrophages present reversible changes in their functional phenotypes and distribution in response to different microenvironmental stimuli, including various cytokines and locally derived molecules, which are tissue- and tumor-specific (Stout et al., 2005; Okabe and Medzhitov, 2014; Ostuni et al., 2015; Kim and Bae, 2016). Therefore, in different histotypes of tumors (Zhang et al., 2014; Cassetta et al., 2019) and different microregions of the same tumor (Mantovani et al., 2002; Kim and Bae, 2016; Yang M. et al., 2018), there can be TAMs with different extent of infiltration and functional status.

In ovarian cancer, Zhang et al. (2014) found the density and the cancer islet/stroma ratio of TAMs vary among serous, mucinous, endometrioid, clear cell and undifferentiated histotypes. In the stroma and lumina of a small number of patient ovarian tumor samples, limited frequencies of iNOS expressing TAMs were found, which were thought to be cytotoxic (Klimp et al., 2001); in contrast, in the malignant ascites of ovarian cancer, abundant TAMs can be found, which are primarily M2-like with pro-tumor capacity (Gupta et al., 2018). As the tumor grows, stimuli in the TME alter, resulting in changes in TAM infiltration and polarization in a tumor progression level-dependent manner. In ovarian cancer studies, TAM and M2 macrophage density were found to increase as cancer stage and ascites volume increased or as lymphatic invasion appeared (Zhang et al., 2014; Ke et al., 2016; Yuan et al., 2017; Gupta et al., 2018); contrarily, M1/M2 ratio decreased as cancer stage increased (Zhang et al., 2014).

Despite expressing similar markers, TAMs may not always have similar functional implications. In colon cancer study, TAMs expressing PD-1 presented weakened phagocytic potency, associating with reduced survival (Gordon et al., 2017), while in early lung cancer study the PD-1+ TAMs did not affect tumor-specific T cell attack against tumor (Singhal et al., 2019). This indicates the necessity of future studies focusing on TAM functional status in the context of tumor tissue types and stages of the disease; this is especially true with ovarian cancer as it has many histotypes and high heterogeneity.

Several studies revealed the prognostic value of TAMs in ovarian cancer. The M1/M2 and M2/TAM ratio have been reported to be positively associated with PFS and OS, while the overall TAM density in ovarian tumors indicated no prognostic significance (Lan et al., 2013; Zhang et al., 2014; Yuan et al., 2017). M2 density in the ascites or tumor samples is associated with reduced relapse-free survival (Reinartz et al., 2014) and PFS (Lan et al., 2013; Yuan et al., 2017). However, there is a controversy in the relationship between M2 density alone and OS: Lan et al. (2013) reported a negative association between the two factors, while Zhang et al. (2014) found no significant relevance. This may be due to the difference of included tumor histotypes.

#### Dendritic Cells

Dendritic cells (DCs) capture endogenous or exogenous antigens, process them, and present the antigenic peptides to other immune cells (Banchereau et al., 2000), acting as a bridge connecting the innate and the adaptive immune system (Timmerman and Levy, 1999; Riboldi et al., 2005). There are two main subtypes of DCs: the conventional DC (cDC) that is specialized in antigen presentation, and the plasmacytoid DC (pDC) that produces IFN upon antigen stimulation aside from activating lymphocytes and other myeloid cells (Labidi-Galy et al., 2011; Vu Manh et al., 2015). cDCs comprise 5–10% of myeloid cells in most tumors; pDCs are rare in mouse tumors but found in most human tumors (Tang et al., 2017).

DCs play key roles in anti-tumor immunity because it is indispensable for T cell immune responses against tumors (Casey et al., 2015). DCs are responsible for tumor antigen recognition, which is the initiating event of the tumor-specific adaptive immune response. In both the animal ovarian cancer model and human HGSOC patients, DCs can sense damage-associated molecular patterns (DAMPs) released from dead cancer cells, such as double-stranded DNA (dsDNA) fragments and calreticulin, an endoplasmic reticulum (ER) chaperone, eliciting Th1 polarized immunity (Ding et al., 2018; Kasikova et al., 2019).

After capturing antigens, DCs present peptides processed from those antigens to CD4+ and CD8+ T cells via major histocompatibility complex class II (MHC II) and MHC I molecules respectively, which subsequently initiate a series of T cell activity (Dudek et al., 2013; Sabado et al., 2017). This process has been reported to be significant for tumor development prevention (MacKie et al., 2003; Galon et al., 2006).

Besides T cell activation, DCs are also crucial for the augmentation of cytotoxic T lymphocytes (CTLs) population in the TME. It is reported that intratumoral cDCs are responsible for intratumoral CTL proliferation both *in vivo* and *in vitro* (Diao et al., 2011), and they are the only group of phagocytosing tumor myeloid cells that can stimulate CD8+ T cell proliferation (Broz et al., 2014). As the major determinant of success in tumor deterrent, from the immune aspect (Budhu et al., 2010), is to increase the functional tumor-infiltrated CTL population, the significance of cDCs in the TME for anti-tumor responses is self-evident.

Effective T cell activation by DCs require DC maturation, a process happens after DC exposing to antigen, characterized by increased membrane expression of MHC and co-stimulatory molecules (CD80, CD86, CD40) (Bol et al., 2016; Bhatia et al., 2019), alteration of chemokine receptors to favor DC lymph node (LN) migration (Drakes and Stiff, 2018); mature DCs produce cytokines that favor Th1 (anti-tumor) immunity. Truxova et al. (2018) found in cohorts of HGSOC patients that tumor-infiltrated mature LAMP+ DCs is robustly associated with Th1 immune responses, clinically favorable cytotoxic activities in the TME and favorable OS.

The process of DC maturation can be hampered by multiple factors, leaving DC immatured, potentially developing into a tolerogenic status and promote immune tolerance (Dhodapkar et al., 2001). Immature DCs express low levels of co-stimulatory molecules and cytokines and mount limited immune activities (Drakes and Stiff, 2018). Factors that lead to DC dysfunction, including the inhibition of DC maturation, involve the immune-modulating molecules in the TME, such as IL-6, IL-10 and VEGF, tumor-derived soluble mediators and exosomes, the activation of oncogene STAT3 in DCs, the ER stress response, and the abnormal intracellular lipid accumulation (Cubillos-Ruiz et al., 2015; Tang et al., 2017; DeVito et al., 2019). These factors suppress DC functions by reducing the expression of co-stimulatory molecules and the secretion of pro-inflammatory cytokines, inhibiting DC lymph node chemotaxis, dampening DC differentiation, inducing tolerogenic phenotypes on DCs and shortening the lifespan of DCs (Tang et al., 2017).

Tolerogenic DCs suppresses anti-tumor immunity via several mechanisms. First, they produce less pro-inflammatory cytokines and induce immune suppressive cytokines. Labidi-Galy et al. (2011) found in a cohort of 44 ovarian cancer patients that intra-tumoural tolerogenic pDCs secreted fewer IFN-α, TNF-α, IL-6, macrophage inflammatory protein-1β and CCL5, while induced IL-10 from CD4+ T cells, promoting immune tolerance in these patients. Second, they harbor enzymes negatively regulating T effector cell functions, such as nitric oxide synthase (NOS) and Indoleamine 2,3-Dioxygenase (IDO) (Casey et al., 2015). IDO is an enzyme catalyzing tryptophan degradation, capable of suppressing tumor-infiltrated lymphocyte proliferation, promoting Treg differentiation, inducing T cell anergy, and promoting tumor angiogenesis as well as metastasis (Munn et al., 2005; Tanizaki et al., 2014; Munn and Mellor, 2016). In EOC patients, there was significantly increased frequency of IDO+ DCs in tumor draining LN compared to the normal donor LN; besides, *in vitro* study revealed IDO significantly inhibited proliferation of tumor-associated lymphocytes derived from EOC patients (Qian et al., 2009).

Many factors are affecting the actual DC functions and behaviors, which are with high plasticity, contributing to either pro-tumor or anti-tumor effect. Tumor expressing molecules are associated with mature DC infiltration. Recently, MacGregor et al. (2019a) found higher surface expression of B7-H4, a B7 family molecule, was correlated with higher mature DC (CD11c+HLA-DRhigh) infiltration in EOC patient samples, which may be associated with increased expression of CXCL17, a monocyte and DC chemoattractant in those tumors. This group have also found that tumour-to-stroma ratio (TSR), which represents the percentage of malignant cell component relative to the stroma in the tumor tissue, have an impact on infiltrated DC phenotype: high TSR was associated with elevated PD-L1 expression on mature DCs (CD11c+HLA-DRhigh) infiltrating in ovarian tumor tissue (MacGregor et al., 2019b).

DC functions can be regulated by their interactions with the proximal milieu, so different locations of DCs may result in different function. Labidi-Galy et al. (2011) discovered that in ovarian cancer patients, tumor pDCs produced less pro-inflammatory cytokines than pDCs from ascites or peripheral blood.

Also, DC performance can vary by different tumor development stage. In an ovarian cancer mouse model, at the early stage, tumor growth was prevented by infiltrating DCs and DC depletion at this stage accelerated tumor expansion; at the advanced stage, however, DCs become immunosuppressive in the TME, abrogating enduring activity of anti-tumor T cells, and DC depletion at this stage significantly delayed disease progression (Scarlett et al., 2012). Similarly, in a mouse model of ovarian cancer, Krempski et al. (2011) also found progressively gained immunosuppressive phenotype of infiltrating DCs as the tumor progressed over time, represented by gradually increased PD-1 expression.

More studies are favored in the future to reveal facts on how DCs functions are regulated, thereby providing clues for therapeutic strategies in maintaining their anti-tumor potential.

#### Myeloid-Derived Suppressor Cells

Myeloid-derived suppressor cells (MDSCs) are a heterogeneous population of myeloid cells that co-express the myeloid surface markers GR-1 and CD11b (Atretkhany and Drutskaya, 2016). MDSCs consist of three phenotypes: PMN-MDSC, M-MDSC and a small group of cells that have myeloid colony-forming activity, including myeloid progenitors and precursors (Gabrilovich, 2017). PMN-MDSCs are similar to neutrophils in phenotype and morphology and represent over 80% of MDSCs, while M-MDSCs are similar to monocyte (Gabrilovich, 2017). Studies have confirmed that MDSCs promote tumor progression by various mechanisms. First, MDSCs are implicated in immune suppression (Ostrand-Rosenberg and Fenselau, 2018). Despite their involvement in the inhibition of many cells in the immune system, MDSCs mainly target T cells. We summarized the mechanisms involved in immune suppression. (1) MDSCs accelerate lymphocyte nutrient depletion (Rodriguez et al., 2004; Srivastava et al., 2010). Both L-arginine and L-cysteine are essential amino acids that are important for T cell activation and function. MDSCs produce ARG1 and depletion of L-arginine through an ARG1-dependent manner (Rodriguez et al., 2004). MDSCs also sequester L-cysteine (Srivastava et al., 2010). Therefore, the amount of ζ-chain in the TCR complex is downregulated, and the proliferation of antigen-activated T cells is suppressed. (2) MDSCs disturb lymphocyte trafficking and viability (Hanson et al., 2009; Sakuishi et al., 2011). Galectin 9, which is expressed in MDSCs, binds to TIM3 on lymphocytes, which induces the apoptosis of T cells (Sakuishi et al., 2011). Similarly, MDSCs express ADAM17, which can decrease the L-selectin level on T cells and limit T cell recruitment in lymph nodes (Hanson et al., 2009). (3) MDSCs promote Treg cell activation and expansion (Gabrilovich et al., 2012). MDSCs stimulate CD4+ T cells to translate into induced Treg (iTreg) cells and expand natural Treg (nTreg) cells. These processes are associated with CD40-CD40L interactions, IFN-γ, IL-10, and TGFβ. (4) MDSCs stimulate the generation of oxidative stress. Oxidative stress is linked to ROS and RNS (reactive nitrogen species) (Gabrilovich, 2017). Superoxide reacts with NO and generates PNT (peroxynitrite), which nitrates T-cell receptors and limits the response of antigen-MHC complexes, thus suppressing T cells directly. PNT also nitrates T-cell-specific chemokines, which decreases the combination of antigenic peptides to MHC and limits the migration of T cells (Molon et al., 2011).

Moreover, MDSCs facilitate neovascularization through different mechanisms. Hypoxia in tumors induces MDSCs to produce VEGF, FGF2 and MMP9. Interestingly, the activation of STAT3 in MDSCs also stimulates neovascularization through IL-1β, CCL2 and CXCL2 release (Bruno et al., 2019). Additionally, these factors stimulate invasion and metastasis by producing MMPs (Ostrand-Rosenberg and Fenselau, 2018).

MDSC is an important part of the tumor microenvironment. MDSC promote tumor progression by regulating immune suppression and facilitating neovascularization. Moreover, the different tumor microenvironment is related to different functions and differentiation of MDSC. Nevertheless, the mechanism is still not clear.

#### Lymphocytes

Lymphocytes, a major component of the TME, include B lymphocytes and T lymphocytes and mediate innate and adaptive immunity, respectively (Sadelain et al., 2017). B lymphocytes accelerate tumor progression by producing protumorigenic cytokines and regulating the Th1: Th2 ratio (Quail and Joyce, 2013). T lymphocytes, a major component of the TME, are crucial for adaptive immunity (Sadelain et al., 2017). T cells develop in the thymus. Before encountering the initial antigen, T cells are regarded as naïve (TN) cells. After antigen encounter, naïve (TN) cells are activated and start differentiation (Smith-Garvin et al., 2009). They proliferate rapidly and release inflammatory cytotoxic granules and cytokines, which activate the immune response. According to the cytokine environment, T cells differentiate into various subsets (Wang M. et al., 2017).

Due to the exclusive expression of CD4 or CD8 markers, mature T cells are categorized into CD3+CD4+, CD3+CD8+ T cells and CD4+ Treg cells (Kishton et al., 2017). CD3+CD4+ T cells are also called helper T cells (Th cells) and regulate immune responses by releasing cytokines that promote or inhibit inflammation (Joyce and Pollard, 2009). CD3+CD4+ T cells can be divided into Th1 and Th2 cells. Among them, Th1 cells produce and release pro-inflammatory cytokines and assist CD3+CD8+ T cells in tumor rejection. Therefore, Th1 cells are antitumorigenic. However, Th2 cells release anti-inflammatory cytokines and promote tumor progression (Joyce and Pollard, 2009; Quail and Joyce, 2013). CD3+CD8+ T cells, called cytotoxic T lymphocytes (CTLs), produce inflammatory cytokines and cell lytic molecules such as perforin and granzyme, which specifically recognize and destroy pathogen-infected or malignant cells (Joyce and Pollard, 2009; Zhang and Bevan, 2011).

Treg cells (CD4+CD25+Foxp3+) also play a crucial role in the immune response (Patsoukis et al., 2016). During development in the thymus, Treg cells universally express Foxp3, representing 5–10% of CD4+ T cells. When responding to TCR and TGF-β, Treg cells show suppression. Treg cells protect hosts against autoimmune diseases through inhibiting self and autoreactive cells (de Aquino et al., 2015). Additionally, Treg cells play a tumourigenic role mainly through immunosuppression monitoring (Lindau et al., 2013). Treg cells regulate the immune response through four mechanisms (Vignali et al., 2008; Facciabene et al., 2012): (1) Secreting immunosuppressive molecules. Treg cells suppress effector T cell functions by secreting cytokines such as IL-10, IL-35, and TGFβ. Additionally, IL-10 and TGFβ are reported as key mediators that limit antitumor immunity and promote tumor progression (Facciabene et al., 2012). Interestingly, these cytokines not only inhibit the function of effector cells but also promote DC polarization to tolerogenic phenotypes. Additionally, Treg cells secrete VEGF, which is also an immunosuppressive molecule (Vignali et al., 2008). Through VEGF, Treg cells exert inhibition and regulate the differentiation of DCs. (2) Cytolysis. Treg cells induce the apoptosis of effector cells by secreting granzyme B and perforin (Vignali et al., 2008). (3) Metabolic disruption. Several mechanisms have been reported for the metabolic disruption regulated by Treg cells. However, it is still controversial. Treg cells deplete the local level of IL-2, which causes effector cells to starve and results in the apoptosis of effector cells. Moreover, with the expression of CD73 and CD39, Treg cells catalyze ATP to adenosine, which inhibits the function of effector T cells (Deaglio et al., 2007; Vignali et al., 2008). (4) Modulation of DC maturation and function. CTLA-4 (cytotoxic T-lymphocyte antigen 4) is expressed on Treg cells, and CD80 and CD86 are expressed on DCs. Treg cells induce DCs through CTLA4–CD80/CD86 interactions, which induces the release of IDO (indoleamine 2,3-dioxygenase). IDO expression depletes essential tryptophan and inhibits the function of effector T cells (Fallarino et al., 2003). Furthermore, Treg cells suppress the function of DCs by depleting costimulatory molecules and inhibiting LAG3 (lymphocyte-activation gene 3) binds to MHC class II molecules (Vignali et al., 2008). It has been reported that TLR (Toll-like receptor), GITR (glucocorticoid-induced TNF receptor), CTLA-4, and FR (folate receptor) directly or indirectly regulate the function of Treg cells (Pasare and Medzhitov, 2003; Callahan et al., 2010; de Aquino et al., 2015). TLR activation decreases the suppressive effect of Treg cells partially through IL-6. GITR, a costimulatory molecule, has a high expression level on Treg cells (Pasare and Medzhitov, 2003). Treatment with anti-GITR mAb downregulates the inhibition of Treg cells. Similarly, CTLA-4 and FR4 are expressed on Treg cells. When blocking CTLA-4 or deleting FR4, the inhibition of Treg cells decreased and active Treg cells were depleted (Callahan et al., 2010).

Several researches implied that lymphocytes were correlated with clinical outcomes in ovarian cancer. Plasma cell and B cell infiltration impacted the prognosis of ovarian cancer. CD138 and CD20 are markers for plasma cells and mature B cells, respectively. Lundgren et al. (2016) found that patients with high expression of CD138 and CD20 were related to advanced tumor grade. Additionally, the Kaplan–Meier analysis suggested that high expression of CD138 was linked to worse OS and OCSS (ovarian cancer-specific survival).

Tumor-infiltrating T cells are associated with clinical outcomes in ovarian cancer (Zhang et al., 2003). Through evaluating 186 frozen tissue samples from patients with advanced ovarian cancer, Zhang‘study demonstrated that the 5-year OS rate was higher in patients whose tumors had T cell infiltration compared to survival of patients whose tumors did not have T cell infiltration. They also confirmed that intratumor T cells were significantly associated with delayed relapse (Zhang et al., 2003).

CD8+ T lymphocyte infiltration extended survival time. In Hamanishi’s research, they demonstrated that patients with CD8+ T lymphocyte infiltration had prolonged PFS and OS (Hamanishi et al., 2007). Similarly, Sato et al. (2005) noticed that patients with high percentages of CD8+ T cells had a greater survival rate than that of patients with low percentages (55 months vs. 26 months). Clarke et al. (2009) also reported that in patients with advanced stage, CD8+ T lymphocyte infiltration was linked to increased PFS, OS and disease-specific survival. Interestingly, Ye et al. (2014) observed that CD137, a TNFR-family member, is expressed on both CD4+ and CD8+ T lymphocytes. Patients with CD137 expression had improved survival in ovarian cancer.

In contrast, Treg cell infiltration indicated poor clinical outcomes. In Curiel’s study, they evaluated 104 women with ovarian carcinoma and found that patients with advanced disease stage had a higher percentage of CD4+CD25+FOXP3+ Treg cells. Furthermore, Treg cells in the tumor sites were linked with decreased survival and a high death hazard (Curiel et al., 2004).

Lymphocytes play important roles in both innate immune responses and adaptive immune responses. Different lymphocytes have different functions. In ovarian cancer, specific lymphocytes infiltration is directly related to patient prognosis. At present, the mechanism of lymphocyte regulation is not completely understood, thereby deserving further investigation.

## Novel Molecular Discoveries in Ovarian TME

Despite the advances of immunostaining technology that has provided important biological features in the ovarian cancer TME, it is still underpowered to adequately detail the multi-variant cellular and molecular interactions in it.

**FIGURE 2:**
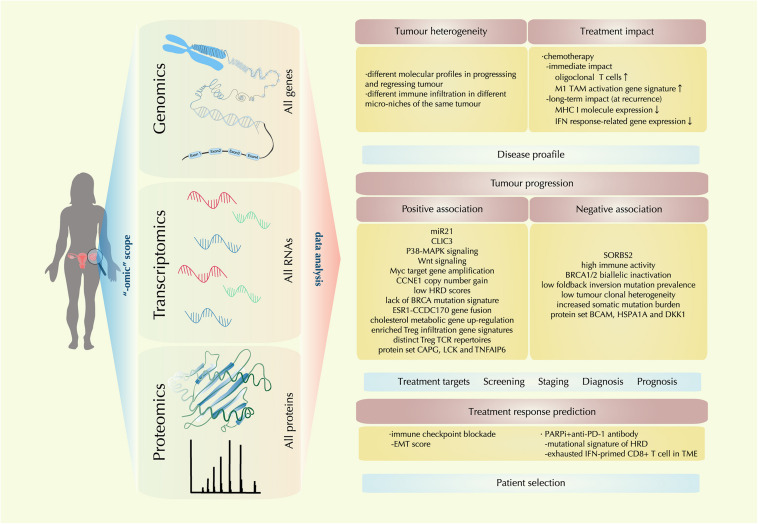
Recent molecular findings by “-omic technology” in the ovarian cancer tumor microenvironment (TME). With the tools of genomic, transcriptomic, proteomic technology, a comprehensive multi-level landscape of ovarian cancer TME is revealed. With data are drawn from the “-omic scope,” the molecular profile of ovarian cancer is further identified in terms of tumor heterogeneity and treatment impact; new discoveries on factors that are either positively or negatively associated with tumor progression are also identified, providing clues for treatment target exploration and novel biomarker designing for disease screening, staging, diagnosis, and prognosis; also, correlations between certain therapeutic regimens and ovarian cancer TME profiles are investigated, providing implication for precision medicine by precise patient selection.

In the past decade, enormous technical development has been realized in sensitive detection and accurate quantification of genomic, transcriptomic, and proteomic features. The “-omic” techniques give scientists a scope from a higher dimension to reveal the TME molecular landscape as a whole. With information from this landscape, multilevel analysis has shed light on the heterogeneous nature of ovarian cancer, the complex and dynamic molecular events in the evolving TME, facilitating the advancement and validation of biomarkers for disease diagnosis, prognosis, treatment targets and treatment response prediction (Figure 2).

Here we review the latest evidence provided by “-omic” technology, revealing the molecular characteristics of ovarian cancer TME in disease development and treatment intervention.

### TME Heterogeneity in Ovarian Cancer

Ovarian cancer is a kind of highly heterogenous tumor; the diversified TME of ovarian cancer is one manifestation for this fact. With immunogenomic approaches, Jiménez-Sánchez et al. (2017) observed changes of different metastatic tumor sites of an HGSOC patient who received multiple times of chemotherapy. They found both progressing and regressing metastases during treatment in the same patient, characterized by immune cell exclusion and T cell infiltration with the oligoclonal expansion of specific subsets respectively. The progressing tumor presented with molecular patterns of immune suppression associated with Wnt signaling and higher HLA mutation and neoepitope loads while regressing sites showed patterns of immune activation with the expression of HLA, IFN-γ, CXCL9 etc. and enriched TCR signaling (Jiménez-Sánchez et al., 2017). By similar approach, recently the same group further discovered co-existence of both immune-cell-excluded and inflammatory microenvironment in the same tumor sites of the same patients with HGSOC, indicating ubiquitous variation in immune cell infiltration even in the micro-niches of the same tumor entity (Jiménez-Sánchez et al., 2020).

### TME Molecular Factors Positively Associated With Tumor Progression

By -omic studies, there is a growing body of evidence unveiling the tumor driving signaling, interactions among cell components and immune profiles in the ovarian cancer TME. Comprehensive analysis of TME cell components, genomic alterations and gene expression revealed that amplification of Myc target genes and Wnt signaling were associated with impaired immune cell infiltration (Jiménez-Sánchez et al., 2020). Using next-generation sequencing technology, Au Yeung et al. (2016) identified high levels of microRNA-21 (miR21) from the exosomes secreted by cancer-associated adipocytes (CAAs) and CAFs, which later were transferred to ovarian cancer cells, suppressing cancer cell apoptosis and conferring chemoresistance. In order to investigate Treg antigen specificity, Ahmadzadeh et al. (2019) adopted TCR β chain deep sequencing in Tregs from multiple tumor samples, including ovarian cancer and discovered that TCR repertoires were distinct from conventional T cells, displaying tumor- and neoantigen-specific reactivity. This finding suggested Tregs clonally expand in an antigen-selective manner in the TME.

Recently, the interplay between metabolism and tumor progression as well as immune suppression among players in the TME has been reported with -omic tools. By phosphoproteomic techniques, Curtis et al. (2019) identified increased activation of the p38-MAPK pathway in CAFs that are co-cultured with ovarian cancer cells and verified that this activation lay the premise for glycogen mobilization in cancer cells, which as an energy source fueled metastatic tumor growth. Another proteomic study identified the central metabolic regulator of CAF, the methyltransferase nicotinamide *N*-methyltransferase (NNMT) being a prominent signature in metastatic stroma tissue of HGSOC patients, which is necessary for the differentiation of competent CAF phenotype, supporting cancer cell migration and proliferation (Eckert et al., 2019). In a mouse model of metastatic ovarian cancer, Goossens et al. (2019) used Gene Ontology (GO) enrichment analysis and found that at a later time after tumor inoculation (day 21), there was an up-regulation of cholesterol metabolic gene clusters in TAMs, resulting in membrane-cholesterol efflux and depletion of lipid rafts from TAMs, leading to IL-4-mediated immune-suppressive TAM reprogramming. All these discoveries provide novel therapeutic targets that could facilitate the current treatment strategy.

There are increasing -omic studies showing an association between TME cellular and molecular profile and patient clinical features. Barnett et al. (2010) earlier have found increased Treg infiltration, represented by several enriched immunologic pathway gene signatures, was associated with higher grade and advanced stage in serous ovarian cancer. From secretome analysis of fibroblasts and cancer cells, Hernandez-Fernaud et al. (2017) identified abundance chloride intracellular channel protein 3 (CLIC3) in the TME of aggressive ovarian cancers, which correlates with poor clinical outcome. Aside from single biomarkers, -omic research enables scientists to discover a correlation between a set of molecular patterns and patient prognosis. With proteomic technology, Finkernagel et al. (2019) identified 779 proteins in the ascites of HGSOC patients and identified protein marker sets to predict patient survival, with CAPG, LCK and TNFAIP6 as the core type 2 signature that has 91.2% correctness in identifying short-term survivors. Another multi-level -omic study with HGSOC primary tumor samples, discovered the association between short-term survival and copy number gain of CCNE1, lack of BRCA mutation signature, low homologous recombination deficiency scores, and the presence of ESR1-CCDC170 gene fusion (Yang S. Y. C. et al., 2018). One study with HGSOC metastatic samples revealed expression pattern of 22 matrisome genes and thereby generated a “matrix index” with their expression; this index was significantly correlated with Treg and Th2 cell signatures and can identify the patient group with shorter OS (Pearce et al., 2018).

### TME Molecular Factors Negatively Associated With Tumor Progression

Together with novel findings of tumor promoting factors, there are also tumor-suppressing molecular patterns uncovered by recent -omic studies. In a study concentrating on RNA binding proteins (RBPs), the authors referred to published literature as well as oncogenic databases and conducted functional verification studies; they identified the sorbin and SH3 domain containing 2 (SORBS2) out of a pool of RBPs, as a suppressor of metastatic colonization of ovarian cancer, which exerted tumor suppressive function by dampening cancer invasiveness and repolarizing MDSCs and TAMs (Zhao L. et al., 2018). Another study investigated the correlation between tumor-infiltrating lymphocytes (TILs) and malignant diversity in HGSOC samples, where the authors found epithelial CD8+ TILs were negatively associated with tumor clonal heterogeneity, suggesting neoantigen-specific depletion of tumor clones and spatial antigen-specific T cell tracking of tumors (Zhang et al., 2018).

Molecular signatures correlating with good clinical prognosis have been reported as well. In Finkernagel et al.’s (2019) ascites proteomic study mentioned above, authors reported markers of BCAM, HSPA1A, and DKK1 as the core type 1 signature with 90.9% of correctness in the identification of long-term survivors. Similarly, in Yang S. Y. C. et al. (2018) study with primary HGSOC samples, they reported increased somatic mutation burden, BRCA1/2 biallelic inactivation, and enriched infiltration of activated as well as memory T cells in long-term survivors. Lastly, in Zhang et al.’s (2018) multi-level study with HGSOC tumor samples, they discovered the combinatorial prognostic value of high immune activity and low mutation prevalence of foldback inversions, which lead to best clinical outcomes.

Detailed mechanistic studies are needed in the future to determine the way of targeting the tumor suppressing molecular patterns that can potentiate therapeutic strategy.

### Treatment Impact and Response Prediction in Ovarian Cancer TME

The tumouricidal treatment causes cell death, incurring multiple changes in the TME. After neoadjuvant chemotherapy (NACT), researchers adopted immunogenic analysis in HGSOC tumors and found increased NK cell infiltration and oligoclonal expansion of T cells, suggesting chemotherapy can potentiate immunogenicity of the primary tumor (Jiménez-Sánchez et al., 2020). Another gene expression analysis revealed ovarian cancer patients treated with paclitaxel had an enriched gene signature linked to M1 TAM activation (Wanderley et al., 2018). However, this immune activation property of chemotherapy may just exist in the early stage right after the treatment; when bulky tumor cells are eradicated, the chemo-resistant cancer stem cells (CSCs) remain and subsequently bring about recurrence. The positively selected CSCs by chemotherapy showed altered lipid metabolism signatures, resulting in accumulation of lactate, which acidifies ascites, leading to T cell dysfunction and Treg polarization (Ahmed et al., 2018). By comparing the gene expression profile of ascites-derived tumor cells from treatment naive (CN) and recurrent (CR) ovarian cancer patients, Ahmed and colleagues found massively reduced MHC I molecule (HLA-C and -B) expression and IFN response-related gene expression, including IFIT2, TMEM173 and MX2 in CR patients, suggesting an immuno-compromised ascites TME in CR after chemotherapy. Hence, CSCs could become a key target in treatment exploitation for CR patients (Ahmed et al., 2018). More comparative studies between CN and CR patients are needed in the future for the discovery of clues that can overcome treatment resistance and targets that can further improve the current regimens.

Due to the heterogeneous nature of ovarian cancer, responses of treatment targeting different tumor driving molecules vary among individuals. With “-omic” tools, the identification of tumor biomarkers that are associated with exceptional clinical response or resistance has become possible. Mak et al. (2016) conducted a genomic and proteomic analysis of EMT signatures in multiple cancers, including ovarian cancer; they identified a set of 77 EMT-related genes, generating an EMT score according to their expression signature; they found EMT score was positively correlated with expression levels of immune checkpoint genes, implicating predictive value of EMT score in treatment response of immune checkpoint blockade. Another study investigated immunogenomic profile in predicting combination treatment response of a PARP inhibitor (PARPi) and an anti-PD-1 antibody in ovarian cancer patients; the authors found two determinants associated with a positive response: mutational signature of defective homologous recombination DNA repair and exhausted CD8+ T cell primed by IFN in the TME. These findings are important for the patient selection of certain treatments, paving the way for future precision medicine.

In conclusion, more mechanistic and phenotypic investigation is required to decipher the roles of certain patterns of molecular alteration in disease development and treatment intervention, so as to facilitate the deployment of more individualized and molecularly informed treatments for ovarian cancer patients.

## Therapeutic Strategies Targeting the TME

### Therapies Targeting CAFs

Several therapies are targeting CAFs in ovarian cancer: (1) direct deletion FAP+ fibroblasts; (2) reverting the activated CAFs into a quiescent state; (3) targeting CAF-specific pathways (Chen and Song, 2019; Barrett and Puré, 2020; Truffi et al., 2020).

#### Direct Deletion FAP+ Fibroblasts

It is well known that CAF has phenotypic heterogeneity. Activated CAFs can selectively express a variety of different biomarkers in specific TMEs environments, such as alpha-SMA FAP, S100A4 and PDGFR (Barrett and Puré, 2020). FAP is a serine protease, which regulates the recruitment, proliferation and differentiation of myofibroblasts. FAP is an important surface marker in CAFs, which exists in more than 90% of CAFs (Chen and Song, 2019). FAP+ cells cannot only promote tumor progression but also block immunotherapy by producing ECM and direct signaling pathways (Puré and Blomberg, 2018). Studies have shown that inhibition of FAP can reduce the infiltration of CAF (Santos et al., 2009). Therefore, targeted therapy for FAP on CAF was proposed.

In Paulette’s research, they examined the gene expression of FAP in high-grade serous EOCs and found that a higher FAP expression in tumor tissue than normal control. They also reported that patients with high FAP expression showed poor OS. Then they blocked the FAP via a FAP-specific siRNA, the results demonstrated that the proliferation of cells was reduced 9–13% (Mhawech-Fauceglia et al., 2015). Thereby, downregulated FAP+ fibroblasts could reduce the proliferation of tumor cells and might be a new treatment for ovarian cancer.

#### Reverting the Activated CAFs Into a Quiescent State

There are two main states of CAFs: quiescent state and active state. In general, most CAFs are in a quiescent state and have low proliferative and metabolic capacity (Hansen et al., 2016; Chen and Song, 2019). However, once the homeostasis is broken, CAFs is activated in order to return to the quiescent state (Hansen et al., 2016). In the tumor microenvironment, not only cancer cells can enhance the activation of CAFs, but some cytokines can also activate CAFs (Chen and Song, 2019). Recent years, some scientists target therapy of CAF by restoring the activation state of tumorigenic CAFs to a static state (Rossmann et al., 1967; Froeling et al., 2011). But it has not been used in ovarian cancer.

#### Targeting CAFs Associated Signal Molecules

Cancer-associated fibroblasts regulate immune cells and ECM via a series of signal molecules. CAFs regulate the function of the myeloid cell and T cell (Barrett and Puré, 2020). CAFs decrease the number of MDSCs through inhibiting CXCL12/CXCR4 signal pathway and promote the differentiation of myeloid cells into DCs via stimulating IL6/STAT3 signal pathway (Gok Yavuz et al., 2019; Truffi et al., 2020). Besides, CAFs inhibit T cells through increase the expression of PD-L1/2, while CAFs activate T cells via stimulating the production of IL-6 (Barnas et al., 2010; Cho et al., 2011). CAFs can active the ECM through secreting growth factors (such as VEGF) and cytokines (such as TGF-β, IL-6 and IL-10) (Kohlhapp et al., 2015).

Recently, several therapies targeting CAFs associated signal molecules have been developed, including TGF-β inhibitors, PDGF inhibitor, Hedgehog inhibitors, FAK inhibitors and IL-6 inhibitors.

TGF-β1 is a cytokine produced by CAFs that plays a significant role in promoting tumorigenesis (Fabregat et al., 2014). A-83-01 is a TGF-β inhibitor. In Yamamura’s research, they found that *in vitro* TGF-β1 treatment stimulated HM-1 cell motility, invasion and adhesion. However, A-83-01 could counteract the effect of TGF-β1. Interestingly, *in vivo*, they found that mice treated with A-83-01 had a longer survival time than that of the control group (Yamamura et al., 2012). Similarly, in Gao’s study, they investigated the tumor-suppressive activity of LY2109761, a TGF-β type I (TβRI) and type II (TβRII) kinase. The results demonstrated that LY2109761 augmented ovarian cancer cell apoptosis. Moreover, combined cisplatin with LY2109761 enhanced the lethal effect of cisplatin in normal and cisplatin-resistant ovarian cancer cells. Furthermore, *in vivo* treatment with cisplatin and LY2109761 reduced the tumor volume in a cisplatin-resistant ovarian cancer model. Therefore, they confirmed that LY2109761 increases the antitumor activity of cisplatin (Gao et al., 2015).

PDGF is a factor that can stimulate other cell trans-differentiation for CAFs. In Matei’s report, they found that PDGFR was expressed in 39% of ovarian cancers. When ovarian cancer cells were treated with imatinib (a PDGFR inhibitor), cells were arrested in the G0–G1 phase. Therefore, they confirmed that imatinib could suppress the proliferation of ovarian cancer cells (Matei et al., 2004). Imatinib also showed its clinical activity on platinum-resistant ovarian carcinoma. In Matei’s study, in patients treated with imatinib mesylate and docetaxel, they found that 1 patient had a complete response and 4 patients had partial responses (ORR: 21.7%) (Matei et al., 2008). Overall, the inhibition of PDGF could suppress tumor proliferation.

Targeting Hedgehog, FAK and IL-6 is also an effective treatment for ovarian cancer. IPI-126 is a Hedgehog inhibitor. When treated with IPI-126, Hh signaling was suppressed, thereby inhibiting the proliferation of serous ovarian cancer (McCann et al., 2011). VS-6063, a FAK inhibitor, suppressed the phosphorylation of FAK. VS-6063 increase the chemosensitivity in taxane-resistant ovarian cancer cells, thus decrease the tumor load (Kang et al., 2013). Tocilizumab is a monoclonal antibody against IL-6R. In a phase I clinical trial, the results showed that patients treated with tocilizumab increase the serum IL-6 and soluble IL-6R. Moreover, increased sIL-6 indicated a longer survival time. Therefore, tocilizumab prolongs survival time in recurrent ovarian cancer (Dijkgraaf et al., 2015).

Cancer-associated fibroblasts is an important component of the tumor microenvironment, which play a pro-tumor function in the process of cancer development, making it a hot spot of targeted therapy. Nevertheless, targeted CAFs therapy still has challenges. Some studies suggest that directly targeted killing of CAF may be a way to reduce CAF infiltration in tumors. But due to the lack of specific cell surface markers, it is hard to precisely target CAFs. The reversal of the functional state of CAF provides a new idea for the development of new anticancer therapies. At present, limiting the function of CAF by targeting stromal CAF signals and effectors has become an important supplement to tumor therapy, but further mechanism and function studies are still needed.

### Anti-angiogenesis Therapy

VEGF is the most typical activator of angiogenesis. Currently, anti-angiogenesis therapy is divided into three types (Cortez et al., 2018; Hironaka, 2019): (1) inhibiting VEGF; (2) inhibiting its receptor, VEGFR; and (3) inhibiting Angs (Table 1).

**TABLE 1:** Clinical trials of therapies that target angiogenesis in ovarian cancer.

Immunotherapy agents	Target	Trial type	Disease status	*N*	Trial number	References
**VEGF inhibitors**						
Bevacizumab	VEGF	Phase 3	Stage III or stage IV epithelial ovarian cancer	1873	NCT00262847	Burger et al., 2011
		Phase 3	Ovarian cancer	1528	ISRCTN91273375	Perren et al., 2011
		Phase 3	Ovarian cancer	1528	ISRCTN91273375	Oza et al., 2015
		Phase 2	Platinum-resistance EOC	44	NCT00097019	Oza et al., 2015
		Phase 3	Platinum-sensitive relapsed epithelial ovarian cancer	484	NCT00434642	Aghajanian et al., 2012
		Phase 3	Recurrent platinum-sensitive ROC	484	NCT00434642	Aghajanian et al., 2015
		Phase 2	Persistent or relapse epithelial ovarian cancer	62	–	Burger et al., 2007
		Phase 3	Relapsed platinum-sensitive epithelial ovarian cancer	674	NCT00565851	Coleman et al., 2017
		Phase 3	Relapsed platinum-resistant ovarian cancer	361	NCT00976911	Pujade-Lauraine et al., 2014
		Phase 2	Relapsed ovarian cancer	70	NCT00072566	Garcia et al., 2008
**VEGF receptors inhibitors**						
Sorafenib	VEGFR	Phase 2	Platinum-resistant ovarian cancer	174	NCT01047891	Chekerov et al., 2018
		Phase 2	Relapsed ovarian carcinoma	71	NCT00093626	Matei et al., 2011
Sunitinib	VEGFR	Phase 2	Platinum resistant ovarian cancer	73	NCT00543049	Baumann et al., 2012
		Phase 2	Recurrent epithelial ovarian cancer	30	NCT00388037	Biagi et al., 2011
Pazopanib	VEGFR	Phase 2	Ovarian cancer	940	NCT00866697	du Bois et al., 2014
		Phase 2	Platinum-refractory or platinum-resistant advanced ovarian carcinoma	74	NCT01644825	Pignata et al., 2015
Nintedanib	VEGFR	Phase 3	Advanced ovarian cancer	1366	NCT01015118	du Bois et al., 2016
Cediranib	VEGFR	Phase 3	Recurrent platinum-sensitive ovarian carcinoma	456	NCT00532194	Ledermann et al., 2016
**Angs inhibitors**						
Trebananib	Ang1 and Ang2	Phase 3	Recurrent ovarian cancer	919	NCT01204749	Monk et al., 2014

#### Anti-VEGF

Bevacizumab is a humanized VEGF monoclonal antibody. It can inhibit ECs proliferation and activation by binding and inactivating VEGF (Eskander and Randall, 2011). Studies have reported that bevacizumab treatment of murine ovarian cancer models could suppress tumor proliferation and prolong survival time (Hu et al., 2002; Monk et al., 2006; Huynh et al., 2007; Mabuchi et al., 2008). Currently, using bevacizumab alone or combined with other therapeutics has had successful outcomes. Bevacizumab was quickly used in the clinic due to its amazing therapeutic effects in animal models. Bevacizumab, as the first FDA-approved anti-angiogenesis antibody, was applied to the treatment of ovarian cancer. Burger et al. (2011) designed a phase 3 trial (GOG-218) to explore the antitumor efficiency of bevacizumab in ovarian cancer. They enrolled 1873 women with newly diagnosed stage III or IV EOC. They randomly divided patients into three groups: control treatment, bevacizumab-initiation treatment, and bevacizumab-throughout treatment. The results demonstrated that the median PFS with the control treatment was 10.3 months, while with bevacizumab-initiation treatment and bevacizumab-throughout treatment, it was 11.2 and 14.1 months, respectively. Additionally, the median OS in the control treatment, bevacizumab-initiation treatment and bevacizumab-throughout treatment was 39.3, 38.7, and 39.7 months, respectively. Thereby, ovarian cancer patients treated with bevacizumab was supposed to have a longer PFS.

Subsequently, the ICON7 trial reported the similarity results in ovarian cancer patients. In the ICON7 trial, they enrolled 1528 patients with ovarian carcinoma and randomly allocated them into the standard-therapy group and bevacizumab group. The data indicated that when the follow-up was 19.4 months, patients in the bevacizumab group had a longer median PFS (restricted mean) than that of the standard-therapy group (21.8 months vs. 20.3 months). Moreover, when prolonging the follow-up to 42 months, the PFS (restricted mean) was 24.1 months in the bevacizumab group and 22.4 months in the standard-therapy group. Additionally, patients in the bevacizumab group had a median OS of 36.6 months compared with a median survival of 28.8 months in the standard-therapy group (Perren et al., 2011). Then, Oza et al. (2015) reported the final OS results of ICON7. They found no significant difference in restricted mean OS between the two groups (45.5 months in the bevacizumab group versus 44.6 months in the standard-therapy group). However, for patients with high risk, the restricted mean OS was longer in the bevacizumab group compared to that in the standard-therapy group (39.9 months in the bevacizumab group vs 34.5 months in the standard-therapy group). For patients with non-high risk, there was no significant difference in the restricted mean OS between the bevacizumab group and the standard-therapy group (49.7 months vs. 48.4 months). Overall, both the GOG-218 and ICON7 trial proved that chemotherapy combined with bevacizumab could not prolong the overall survival in ovarian cancer. However, high risk patients treated with chemotherapy plus bevacizumab show an OS benefit compared to those treated chemotherapy alone.

Bevacizumab also showed antitumor activity in platinum-resistant EOC. Cannistra et al. (2007) designed a phase 2 trial for bevacizumab in patients with platinum-resistant EOC or peritoneal serous cancer. They enrolled 44 patients and treated them with intravenous bevacizumab 15 mg/kg every 3 weeks. In the research, seven patients had a partial response. Patients had a median PFS of 4.4 months. So in platinum-resistant cancer, bevacizumab also had antitumor activity.

Bevacizumab also has antitumor activity in recurrent ovarian cancer. Aghajanian et al. (2012) reported a phase 3 clinical trial (OCEANS) in platinum-sensitive relapsed ovarian cancer. In total, 484 patients were enrolled and randomly assigned to the bevacizumab arm (*n* = 242) or placebo arm (*n* = 242). All patients were treated with carboplatin. The results showed that patients in the bevacizumab arm had a longer PFS compared to that of patients in the placebo arm (12.4 months vs. 8.4 months, respectively). Additionally, patients in the bevacizumab arm also showed an enhanced objective response rate (78.5% versus 57.4%) and duration of response (10.4 months versus 7.4 months) compared with those of patients in the placebo arm. They also analyzed the OS of the two arms. However, the results showed no significant difference in OS between the bevacizumab arm and the placebo arm (33.6 months in the bevacizumab arm vs 32.9 months in the placebo arm) (Aghajanian et al., 2015). Later, Coleman et al. (2017) reported a phase III clinical trial of bevacizumab combined with chemotherapy in patients with relapsed platinum-sensitive EOC. The data showed that the median OS was significantly longer in the chemotherapy plus bevacizumab group compared to that of the chemotherapy group (42.2 months in the chemotherapy plus bevacizumab group versus 37.3 months in the chemotherapy group). Additionally, the median PFS in patients with chemotherapy was 10.4 months, while in chemotherapy plus bevacizumab, the median PFS was 13.8 months (Coleman et al., 2017). Above studies implied that bevacizumab could improve the PFS and median OS in recurrent platinum-sensitive recurrent ovarian cancer, while it had no significant effect for OS.

There have also been studies using bevacizumab-treated relapsed platinum-resistant ovarian cancer. In a phase III clinical trial (AURELA) the results demonstrated that patients in the bevacizumab with chemotherapy group had a significantly increased PFS rate than that of the chemotherapy group. The median PFS in the bevacizumab with chemotherapy group and the chemotherapy group was 6.7 and 3.4 months, respectively. However, there was no meaningful OS increase in the bevacizumab with chemotherapy group. Therefore, they thought that the combination of bevacizumab with chemotherapy could improve the PFS in patients with relapsed platinum-resistant ovarian cancer (Pujade-Lauraine et al., 2014).

Bevacizumab combined with cyclophosphamide also showed its effect in recurrent ovarian cancer. In a phase 2 clinical trial, seventy patients with relapsed ovarian carcinoma were treated intravenously with bevacizumab and cyclophosphamide. The results showed that 17 patients had a partial response. Moreover, patients had a median progression time of 7.2 months and a median survival time of 16.9 months (Garcia et al., 2008).

VEGF plays a crucial role in angiogenesis. Nowadays, due to the pro-tumor effect in tumor progress, targeting VEGF became an attractive therapeutic method. As the first FDA-approved anti-angiogenesis antibody, bevacizumab was applied to the treatment of cancer. Studies demonstrated that bevacizumab combined with chemotherapy increases the PFS in newly prognosis ovarian cancer, platinum-resist ovarian cancer and recurrent ovarian cancer. Nevertheless, bevacizumab added to chemotherapy could not enhance the OS. Further investigated was needed.

#### Inhibitors of VEGF Receptors

Sorafenib (also called BAY 43-9006) is a double inhibitor of VEGF and RAF kinase that can promote tumor angiogenesis by targeting RTKs and the PAF/MEK/ERK pathway (Wilhelm et al., 2004). Chekerov et al. (2018) reported a randomized, double-blind, placebo-controlled, phase II study of the effect of sorafenib in combination with topotecan in women with platinum-resistant ovarian carcinoma. They randomly assigned patients into the topotecan combined sorafenib (oral 400 mg bid) group (*n* = 85) of the topotecan plus placebo group (*n* = 89). The results showed that patients in the topotecan plus sorafenib group had improved PFS compared with that of the topotecan plus placebo group (6.7 months vs. 4.4 months). There were the same results for OS (17.1 months in the topotecan plus sorafenib group vs. 10.1 months in the topotecan plus placebo group). Subsequently, Matei et al. (2011) described a phase 2 clinical trial of sorafenib in relapsed ovarian carcinoma. They treated patients with oral 400 mg sorafenib bid. The results suggested that only 2 women had partial response and 20 patients had stable disease. In brief, sorafenib could increase the survival time in ovarian cancer.

Sunitinib is a multi-targeted tyrosine kinase inhibitor that acts through targeting PDGF receptor (PDGFRs), VEGFR-1-2-3, KIT (a stem cell factor) and Flt3 (a tyrosine protein receptor) (Bauerschlag et al., 2010). Baumann et al. (2012) reported a phase II trial of sunitinib in patients with platinum-resistant ovarian cancer. Patients were divided into a non-continuous sunitinib group and a continuous sunitinib group. Six patients in the non-continuous group and two patients in the continuous group had a complete response or partial response. Additionally, the median PFS times for the non-continuous group and continuous group were 4.8 and 2.9 months, respectively. The median OS was 13.6 months vs. 13.7 months for the non-continuous group and continuous group. Additionally, Biagi et al. (2011) reported the effect of sunitinib in relapsed ovarian cancer. In patients who received oral sunitinib treatment, there was a median PFS of 4.1 months. Hence, using sunitinib in recurrent ovarian cancer patients could increase the survival time.

Pazopanib was an anti-angiogenic drug that targets VEGF receptor, FGF receptors (FGFRs) 1–3, and PDGFRs α and β. A phase 2 enrolled 940 patients with ovarian cancer and randomly divided into a pazopanib group (800 mg once daily for 24 months) (*n* = 472) and a placebo group (*n* = 478). The results revealed that patients in the pazopanib group had an improved median PFS compared with that of the placebo group (mean PFS 17.9 months versus 12.3 months). Conversely, there was no difference in OS between the two groups. Additionally, Pignata et al. (2015) reported a phase 2 trial to assess the effect of combined pazopanib and paclitaxel in patients with platinum-refractory or platinum-resistant advanced ovarian carcinoma. They enrolled 74 patients who were randomly assigned to the paclitaxel and pazopanib group (*n* = 37) or the paclitaxel group (*n* = 37). The data indicated that patients in the paclitaxel and pazopanib group had significantly longer PFS than that of patients in the paclitaxel group (mean PFS 6.35 vs 3.49 months). The median OS was 19.1 months in the paclitaxel and pazopanib group and 13.7 months in the paclitaxel group. Above researches indicated that pazopanib treatment had an improve PFS in ovarian cancer.

Nintedanib is also an oral angiokinase inhibitor, which can inhibit the effect of VEGFR1-3, FGRs 1-3 and PDGFRs α and β. A phase 3 trial was performed to elucidate the effect of nintedanib combined with first-line chemotherapy in advanced ovarian cancer. Of the 1366 patients enrolled, 911 were assigned to the nintedanib group, and 455 were assigned to the placebo group. The data suggested the mean PFS was 17.2 months in the nintedanib group compared with 16.6 months in the placebo group. Therefore, they suggested that the combination of nintedanib and first-line chemotherapy could significantly increase the PFS in advanced ovarian cancer.

Cediranib is a VEGF receptor (VEGFR1-3) inhibitor. In a randomized phase 3 trial (ICON6) the investigators randomly assigned 456 patients with relapsed platinum-sensitive ovarian carcinoma into three groups: group A received placebo with chemotherapy and then placebo maintenance, group B received cediranib 20 mg with chemotherapy and then placebo maintenance, and group C received cediranib once daily with chemotherapy and then cediranib once daily maintenance. The results showed that 90% of patients (410 of 456) had disease progression, including 96% of patients in group A (113 of 118), 90% of patients in group B (156 of 174) and 86% of patients in group C (141 of 164). Moreover, patients in group C had a longer median PFS compared to that of groups A and B (mean PFS = 8.7, 9.9, and 11.0 months in groups A, B, and C, respectively) (Ledermann et al., 2016). Thus, they considered cediranib with chemotherapy could significantly improve the PFS in patients with relapsed ovarian cancer.

VEGFR inhibition is an important component of anti-angiogenic therapy. Several VEGFR inhibitors have been introduced into clinical studies. The research results suggest that treatment of VEGFR inhibitors could improve the PFS in ovarian cancer. These provide a novel therapeutic option for ovarian cancer.

#### Angs Inhibitor

Ang1 and Ang2, expressed on ECs, were connected with the TIE2 receptor. They could mediate vascular remodeling by a signaling pathway, which was different from the VEGF pathway. Trebananib (also called AMG386) is a peptide-Fc. Through binding with Ang1 and Ang2, it prevents the connection of the TIE2 receptor and Angs. Thus, trebananib showed antitumor activity in ovarian cancer. A double blind phase 3 study detected the antitumor activity of trebananib in patients with recurrent ovarian cancer. A total of 919 patients were enrolled, and they were randomly divided into the trebananib group (*n* = 461) and the placebo group (*n* = 458). In the placebo group, patients were treated with intravenous paclitaxel 80 mg/m2 and placebo weekly. The results showed that patients with trebananib treatment had a significantly longer median PFS compared to patients with placebo treatment (7.2 months compared to 5.4 months). However, there were no significant differences between the trebananib group and the placebo group in the OS analysis (17.3 months in the paclitaxel group and 19.0 months in the placebo group) (Monk et al., 2014).

Trebananib inhibited Angs 1 and 2 and improved the PFS in ovarian cancer. However, the role of Angs inhibitors in recurrent ovarian cancer remains to be further studied.

### TAM-Targeted Antitumor Strategies

Tumor-associated macrophages are a crucial part of the TME. It is known that TAMs have a significant association with the proliferation, invasion, migration and clinical outcomes of ovarian carcinoma. In recent years, significant progress has been made in research on TAM-targeted strategies. Based on previous research, TAM-targeted strategies can be divided into four types: (1) suppressing macrophage recruitment; (2) inhibiting TAM survival; (3) increasing the tumouricidal activity of M1 macrophages; and (4) limiting the tumor-promoting activity of M2 macrophages (Tang et al., 2013; Cassetta and Pollard, 2018).

#### Suppressing Macrophage Recruitment

Various chemokines and cytokines promote macrophage recruitment to tumor tissues, such as C-C motif chemokine ligand 2 (CCL2), macrophage colony-stimulating factor (GM-CSF), VEGF, CXCL-12 and hypoxia-inducible factors (HIFs) (Tang et al., 2013; Cassetta and Pollard, 2018). Thus, regulating the relevant chemoattractants is a promising approach for suppressing macrophage recruitment and tumor therapy.

CCL2 (also called monocyte chemotactic protein-1 [MCP-1]) is a member of the MCP chemokine family that is produced by tumor cells and stromal cells such as myeloid cells, ECs and fibroblasts and acts as a chemoattractant for T cells, NK cells and monocytes (Ueno et al., 2000; Conti and Rollins, 2004). CCR2 is a receptor of CCL2, including CCR2A and CCR2B. Among them, CCA2B is the main isoform of CCR2 that is highly expressed on NK cells and monocytes. CCR2A is expressed on smooth muscle cells and a portion of monocytes. It has been reported that CCL2-CCR2 signaling is involved in tumor metastasis (Lim et al., 2016). In the initial stage of metastasis, tumor cells breakdown ECM and travel to blood vessels. During this stage, CCL2 guides the migration of cancer cells through linking with CCR2. In addition, CCL2 promotes the migration of cancer cells by inducing the expression of MMP2 as well as MMP9 (Tang and Tsai, 2012). Then, cancer cells invade into blood vessels for metastatic dissemination, which requires TAMs. CCL2 promotes cancer cell intravasation and extravasation because it is a chemoattractant for TAMs. Interestingly, CCL2-CCR2 signaling stimulates angiogenic switching via recruiting myeloid cells and suppresses immune-mediated killing by recruiting MDSCs (Huang et al., 2007; Low-Marchelli et al., 2013).

CCL2 was highly expressed on paclitaxel-resistant ovarian cancer cells and showed an antitumor effect in ovarian cancer. Moisan et al. reported C1142 (a mouse CCL2 inhibitor) combined with carboplatin in the treatment of ovarian cancer mouse model could improve the efficacy of carboplatin (Moisan et al., 2014). Additionally, Sandhu et al. (2013) reported a phase I trial investigating the effect of carlumab in solid tumors. They enrolled forty-four patients in total, including eight ovarian cancer patients. All of them received different doses of carlumab (also called CNTO 888), which is a human anti-CCL2 monoclonal antibody. The results showed that patients with advanced ovarian carcinoma achieved a more than 50% decrease in CA125 and achieved 10.5 months of stabilized disease.

M-CSF (also called CSF-1) is also a chemokine for macrophages and is secreted by a variety of stromal cells and epithelial cells (Wyckoff et al., 2004). In addition, its receptor, named M-CSFR, CSF-1R or CD115, is a receptor tyrosine kinase and is restricted to mononuclear phagocytes (Bonelli et al., 2018). It is known that the binding of CSF-1 and CSF-1R regulates the differentiation, function and survival of macrophages through inducing tyrosine kinase (TK)–mediated autophosphorylation in the cytoplasm and the production of intracellular cascade signals (Chitu and Stanley, 2006; Hume and MacDonald, 2012). Hence, the CSF-1/CSF-1R axis can be blocked by anti-CSF antibodies, anti-CSF-1R antibodies and some molecule inhibitors that suppress the function of tyrosine kinases (Hume and MacDonald, 2012; Ries et al., 2015).

Studies implied that CSF-1R inhibitors suppressed the proliferation and metastasis in ovarian cancer via blocking macrophages. Moughon et al. (2015) reported using CSF-1R inhibitors in advanced ovarian carcinoma. They found that during the late stage of ovarian cancer, mice treated with GW2580, a CSF-1R kinase inhibitor, had markedly reduced ascites volume and infiltration of M2 macrophages. Thus, they thought that CSF-1R inhibitors decreased the ascites volume by blocking macrophages. Subsequently, Yu et al. (2018) reported the antitumor effect of CSF-1R inhibitors in cisplatin-resistant ovarian cancer. They found that CSF-1R was highly expressed in cisplatin-resistant SKOV3 and CaoV-3 cells (human ovarian cancer cell lines). When cancer cells were treated with pexidartinib, a CSF-1R inhibitor, with or without cisplatin, the combination of pexidartinib and cisplatin significantly inhibited ovarian cancer cell proliferation and induced apoptosis in cancer cells. They also found that the combination of pexidartinib and cisplatin treatment more efficiently suppressed tumor growth compared to using cisplatin alone in mouse models. Thus, they confirmed that CSF-1R inhibitors could inhibit the proliferation of cisplatin-resistant ovarian cancer. Recently, Lu and Meng (2019) reported the function of BLZ945 in ovarian cancer. They established an ovarian cancer model and treated them with docetaxel with or without BLZ945, which is an inhibitor of the CSF1CSF1R pathway. The data showed that both the docetaxel group and BLZ945 group had decreased tumor growth. Similarly, the docetaxel plus BLZ945 group had a significant decrease in tumor growth compared with that of the docetaxel or BLZ945 alone group. Additionally, docetaxel increased TAM infiltration, while the BLZ945 group showed decreased TAM abundance. They also found that the BLZ945 group had decreased VEGF and MMP9 expression levels, which were closely related to metastasis. Correspondingly, the DTX and BLZ945 combination group had significantly deregulated VEGF and MMP9. Moreover, the docetaxel plus BLZ945 group had less lung metastasis than those observed in the other groups. Therefore, they confirmed that BLZ945 inhibited the proliferation and metastasis of ovarian cancer by suppressing TAMs.

CCL2, M-CSF, VEGF, CXcl-12, and HIFs are molecules that can improve macrophage recruitment. As mentioned above, targeted related chemical attractants enhance the anti-tumor activity of ovarian cancer. Therefore, studies suggested that inhibition of macrophage recruitment can be used as a complement to treatment strategies.

#### Inhibiting TAM Survival

As a part of TAM-targeted strategies for cancer, the inhibition of TAM survival may be realized by attenuated bacteria, immunotoxin-conjugated mAbs and chemical reagents that induce macrophage apoptosis or by activating immune cells such as T lymphocytes to kill TAMs.

Bisphosphonate is an important drug for depleting macrophages. Kobayashi et al. reported that incubating SKOV3 and OVCAR5 cells (human ovarian cancer cell lines) with alendronate a second-generation bisphosphonate, they found that alendronate exerted concentration-dependent growth inhibition on these cells. Then, they treated the mogp-TAg transgenic mouse with alendronate and discovered a significant decrease in tumor mass in the reproductive tract. Additionally, mice that received alendronate treatment tolerated it well and showed no influence on body weight. Therefore, they suggested that alendronate inhibited the proliferation of ovarian cancer *in vivo* and *in vitro* (Kobayashi et al., 2017).

Apart from inducing macrophage apoptosis, TAMs can be suppressed by the activation of an adapted immune response, especially cytotoxic T lymphocytes. However, we did not find studies that applied the inhibition of the adapted immune response to ovarian cancer treatment.

#### Increasing the Tumouricidal Activity of M1 Macrophages

In the TME, immunosuppressive M2 macrophages comprise mainly TAMs (Ren et al., 2014). However, due to the plasticity of polarization, TAMs still maintain the potential to repolarize from tumor-promoting M2 macrophages to tumor-resisting M1 macrophages (Biswas and Mantovani, 2010). As mentioned before, the polarization of macrophages relies on cytokines. When exposed to IFN-γ, LPS, GM-CSF and IL-12, macrophages mainly polarize to M1 macrophages. In contrast, when exposed to IL-4, IL-10, and IL-13, macrophages polarize to M2 macrophages. Therefore, regulating these cytokines could contribute to TAM-targeted antitumor strategies.

It was reported that the NF-κB signaling pathway was associated with TAM polarization (Hagemann et al., 2009; Biswas and Lewis, 2010; Mancino and Lawrence, 2010; Tang et al., 2013).

The NF-κB family includes five transcription factors: RelA/p65, RelB, c-Rel, NF-κB1 (precursor proteins p50/p105) and NF-κB2 (p100/p52) (Hagemann et al., 2009). Several agents, such as TLR (Toll-like receptors) agonists, anti-IL-10R mAb and anti-CD40 mAb, can activate NF-κB through classical or non-classical pathways (Mantovani et al., 2004; Sica and Bronte, 2007; Mancino and Lawrence, 2010). In the NF-κB signaling pathway, NF-κB modulates many crucial genes in macrophages and many tumor-promoting genes, such as VEGF, IL-6, TNF-α, and COX2 (Hagemann et al., 2009; Biswas and Lewis, 2010). In addition, the inactivation of NF-κB mediates polarization of TAMs to immunosuppressive M2 macrophages, while NF-κB reactivation adjusts TAMs to tumouricidal M1 macrophages (Biswas and Lewis, 2010). The NF-κB signaling pathway also regulates the development and proliferation of T and B lymphocytes (Jost and Ruland, 2007), modules angiogenesis and plays a vital role in tumorigenesis (Pramanik et al., 2018).

Recently, a TLR-7 agonist, which can activate NF-κB, was applied to cancer treatment. Geller et al. (2010) reported a clinical trial on the antitumor activity of a TLR-7 agonist in relapsed ovarian, cervix and breast cancer. They enrolled fifteen patients, including ten patients with recurrent ovarian cancer. All of the patients received 852A (a TLR-7 agonist). The results showed that only one patient with stage IIIc serous ovarian cancer had stable disease after treatment with 24 doses of 852A, while she did not continue the trial because of disease progression.

IL-12 is also a factor that can enhance macrophage polarization to M1 macrophages. IL-12 can also promote Th1 response, which polarizes macrophage to M1 macrophages (Biswas et al., 2012; Lu, 2017; Shapouri-Moghaddam et al., 2018). Silver et al. (1999) reported that in an animal model of ovarian cancer, mice treated with IL-12 had decreased tumor growth and even tumor regression. Therefore, they suggested that IL-12 had an antitumor effect on ovarian cancer. Subsequently, a phase I trial reported the treatment of relapsed chemotherapy-resistant ovarian cancer with the IL-12 plasmid/lipopolymer complex. The results showed that 31% of the patients treated with IL-12 treatment had stable disease, while 69% of patients had progressive disease. In addition, patients with high IL-12 treatment had longer survival than that of patients with low IL-12 treatment (Anwer et al., 2010). Similarly, Anwer et al. (2013) reported that 17% of patients with platinum-sensitive relapsed ovarian cancer treated with IL-12 had completed response, 33% had a partial response, 42% had stable disease, and 8% had progressive disease. Later, Alvarez et al. (2014) reported that treated platinum-resistant relapsed ovarian cancer patients with EGEN-001 (an IL-12-based immunotherapeutic agent) no patients had complete or partial responses, 35% (7 of 16 patients) had stable disease, and 45% (9 of 16 patients) had progressive disease. Moreover, the median OS and PFS of EGEN-001-treated patients were 9.17 and 2.89 months, respectively. Therefore, they suggested the limited activity of EGEN-001 in platinum-resistant relapsed ovarian cancer.

Tumor-associated macrophages have the plasticity for polarization, which means that TAMs could repolarization from tumor promoting M2-type to tumor-killing M1-type. Several molecular are reported associated with TAM re-polarization, such as NF-κB and IL-12. Recent years, several researches implied that targeted NF-κB and IL-12 enhance macrophage polarization to M1 macrophages in ovarian cancer. However, the specific signaling pathways are not fully clear. Hence, further studies are needed.

#### Limiting the Tumor-Promoting Activity of M2 Macrophages

Apart from the above TAM-targeted antitumor strategies, limiting the tumor-promoting activity of M2 macrophages is also a promising strategy. STAT3, a member of the STATs (Signal Transducers and Activators of Transcription) family, is generally inactivated and located in the cytoplasm (Laudisi et al., 2018). It can be activated by various receptors, including cytokines such as IL-6 and IL-10, as well as IL-11 and growth factors such as VEGF, EGF and FGF (Chai et al., 2016; Furtek et al., 2016). Once binding to their ligand, the conformation of the receptors changes, which promotes signal propagation and leads to the activation of JAKs. Then, activated JAKs, including JAK1 and JAK2, induce the phosphorylation of STAT3 at Tyr705 and lead to the translocation of activated STAT3 dimers to the nucleus, where it binds to DNA and enhances gene transcription (Yu H. et al., 2014). It has been reported that STAT3 could modulate tumorigenesis by regulating associated gene expression. For instance, STAT3 regulates the expression of c-Myc and cyclin D1, which are linked to the cell cycle (Luwor et al., 2013). STAT3 also modulates angiogenesis via the gene expression of VEGF and IL-8 and regulates migration by MMP-2 and MMP-9 gene expression (Zhang et al., 2010). More recently, studies found that STAT3 is associated with the polarization of TAMs (Tang et al., 2013; Wang et al., 2018). Additionally, the inhibition of STAT3 decreased TAM polarization to M2 macrophages (Fujiwara et al., 2011).

HO-3867 is an inhibitor of STAT3 and has a significant antitumor effect on ovarian cancer (Selvendiran et al., 2010; Tierney et al., 2012; Rath et al., 2014; Tang et al., 2015; Bixel et al., 2017; Saini et al., 2017; Yoshikawa et al., 2018). Selvendiran et al. (2010) found that HO-3867 induced the apoptosis of A2780 cells through activating caspase-3 and caspase-8 and promoted G2/M cell-cycle arrest via regulating cell-cycle regulatory molecules such as cyclin, p21, p27, p53 and cdk2. Additionally, they observed a dose-dependent reduction of tumor volume in mice with ovarian cancer. Then, Rath et al. (2014) reported that HO-3867 reduced tumor growth in a chemotherapy-resistant ovarian cancer model in a dose-dependent manner. Subsequently, Saini et al. (2017) found that HO-3867 inhibited tumor size and tumor metastasis in ovarian cancer.

WP1066 is also a STAT3 inhibitor with antitumor activity. Tang et al. (2015) found that WP1066 markedly suppressed the clonogenicity and invasion activity of SKOV3 and SKOV3/DDP cells (a cisplatin-resistant ovarian cancer cell line). However, it increased the apoptosis of SKOV3 and SKOV3/DDP cells. When treated with WP1066 and cisplatin in combination, the inhibition of proliferation and apoptosis increased compared to that of cisplatin alone. Thus, they suggested that WP1066 inhibited proliferation and metastasis and increased the apoptosis and chemosensitivity of ovarian cancer cells.

M2 macrophages show tumor-promoting activity. Above studies provide that STAT3 is involved in increased M2 macrophages. The inhibition of STAT3 decreased the proliferation and metastasis of cancer cells.

In summary, TAMs-targeted treatment strategies include suppressing macrophage recruitment and TAM survival and increasing the transformation of M2 macrophages to M1 macrophages. TAMs participate in tumor progression through fairly complex mechanisms. However, our understanding of TAMs and its interaction with the tumor microenvironment are not deep enough. Therefore, more researches are needed to facilitate the development and clinical application of TAM-targeted antitumor strategies.

### Immune Checkpoint Inhibitors

Immune checkpoints play important roles in modulating T cell function in the TME (Zhao and Subramanian, 2017). Immune checkpoint therapy limits inhibitory pathways in T cells, thereby enhancing antitumor immune responses (Sharma and Allison, 2015). Hence, it changes cancer treatment. Among them, CTLA-4 and PD1/PD-L1 are important immune checkpoints for ovarian cancer (Mittica et al., 2016; Bose, 2017) (Table 2).

**TABLE 2:** Immune checkpoint inhibitors in ovarian cancer.

Immunotherapy agents	Target	Phase	Disease status	*N*	Trial number	References
**CTLA-4 inhibitors**						
Ipilimumab	CTLA-4	–	Ovarian cancer	2	–	Hodi et al., 2003
		–	Stage IV ovarian cancer	9	–	Hodi et al., 2008
		Phase 2	Recurrent platinum-sensitive ovarian carcinoma	40	NCT01611558	–
**PD-1 inhibitors**						
Nivolumab	PD-1	Phase 2	Platinum-resistant ovarian cancer	20	UMIN000005714	Hamanishi et al., 2015
Pembrolizumab		Phase 1b	Advanced ovarian cancer	26	NCT02054806	Varga et al., 2019
**PD-L1 inhibitors**						
BMS-936559	PD-L1	Phase 1	Ovarian cancer	200	NCT00729664	Brahmer et al., 2012
Avelumab		Phase 1b	Refractory or relapsed ovarian cancer	125	NCT01772004	Disis et al., 2019

#### Anti-CTLA-4 Antibody

CTLA-4 is a transmembrane glycoprotein that is mainly expressed on activated T cells, such as regulatory T cells and memory T cells (Tarhini and Iqbal, 2010). It is widely known that CTLA-4 inhibits the T lymphocyte-mediated antitumor immune response by intrinsic and extrinsic cell pathways (Tarhini and Iqbal, 2010; Brunner-Weinzierl and Rudd, 2018). The intrinsic cell pathways include suppressing cytokine receptor signaling and protein translation, activating ubiquitin ligases and recruiting phosphatases (Tarhini and Iqbal, 2010). In contrast, in the extrinsic cell pathways, CTLA-4 competitively binds to members of the B7 family and transmits inhibitory signals, thereby reducing the activation of T cells (Egen et al., 2002; Zhao Y. et al., 2018). In addition, CTLA-4 produces a reversing signal via B7 and induces the secretion of IDO, resulting in the decomposition of tryptophan and inhibition of the proliferation of T cells (Boasso et al., 2005).

Anti-CTLA-4 antibodies have immune suppression by limiting CTLA-4 to bind with members of the B7 family, thereby suppressing the recognition of antigen-specific T cells. Hodi et al. (2003) reported a trial investigating the activity of MDX-CTLA-4 (a CTLA-4 inhibitor, also called ipilimumab) in patients with metastatic ovarian cancer. One patient with ovarian carcinoma had a stable CA-125 level 1 month after antibody injection. Meanwhile, she experienced a reduction in ascites and pain. The other patient had a 43% decrease in CA-125 level in the initial 2 months. Subsequently, Hodi et al. (2008) treated ovarian cancer patients with ipilimumab. The data showed that one patient had obvious antitumor effects. There is also a phase II clinical trial underway studying the antitumor effect of ipilimumab in patients with relapsed platinum-sensitive ovarian carcinoma.

#### Anti-PD1/PD-L1 Antibodies

PD-1 (also called CD279) is a type I transmembrane protein that is expressed on a variety of immune cells, including activated T cells and B cells, NK cells, monocytes and DCs (Frydenlund and Mahalingam, 2017). PD-L1 and PD-L2 are receptors for PD-1, which all belong to the B7 family (Moy et al., 2017). Among them, PD-L1 shows a wide range of expression on hematopoietic cells and non-hematopoietic cells, while PD-L2 is only expressed on APCs (antigen presenting cells) (Moy et al., 2017). PD-L1 and PD-L2 can be induced by extrinsic proinflammatory signals, such as TNF-α, IFN-γ, ILs and GM-CSF (Wang X. et al., 2017; Zhang et al., 2017; Mimura et al., 2018). Apart from this, PD-L1 is also induced by intrinsic signaling pathways, including the PI3K-AKT pathway and the JAK/STAT pathway (Jiang et al., 2019). It is known that PD-1 has an immune-suppressive ability through binding with PD-1 receptors (Brunner-Weinzierl and Rudd, 2018). After binding with PD-1 receptors, PD-1 stimulates intracellular signaling pathways and suppresses immune cell activation, which inhibits the production of cytokines and antibodies from immune cells, which in turn exhausts the immune cells and maintains immune system homeostasis (McDermott and Atkins, 2013). Additionally, PD-1 can not only inhibit T lymphocyte activation and accelerate T lymphocyte apoptosis but can also be regulated by molecules in the TME, such as TGF-β, IL-7, IL-15, IL-21 and IFN-α (Kinter et al., 2008; Terawaki et al., 2011; Rekik et al., 2015; Jiang et al., 2019). PD-1 inhibitors, including anti-PD-1 antibodies and anti-PD-L1 antibodies, reverse the suppression of antigen-specific T cells through blocking PD-1 or PD-1 ligands (Hamanishi et al., 2016).

As precision medicine develops, patient selection tends to be a social economic trend, which also goes for PD-L1/PD-1 blockade therapy. In ovarian cancer, the expression of PD-L1 on DCs and macrophages correlates with clinical efficacy of PD-L1 and PD-1 blockade, suggesting that the APC PD-L1 expression may be a predicting factor of therapeutic benefit and patient selection indicator (Lin et al., 2018).

##### Anti-PD-1 antibodies

Nivolumab is an anti-PD-1 monoclonal antibody that inhibits the combination of PD-1 ligands. There was a clinical trial exploring the effect of Nivolumab in patients with platinum-resistant ovarian cancer. It divided twenty patients with platinum-resistant ovarian cancer into a high-dose cohort and a low-dose cohort. The results showed that in the low-dose group, one patient had a partial response, and four patients had stable disease. However, in the high-dose group, two patients had a complete response, and two patients had stable disease. Additionally, for all 20 patients, the median PFS time and median overall time were 3.5 and 20.0 months, respectively (Hamanishi et al., 2015).

Pembrolizumab is also a humanized anti-PD-1 monoclonal antibody. Andrea and his colleagues reported that treated advanced ovarian cancer patients with pembrolizumab, One patient with complete response, two patients with partial response, seven patients with stable disease and sixteen patients with disease progression. The median OS and PFS were 13.8 and 1.9 months (Varga et al., 2019), respectively.

##### Anti-PD-L1 antibodies

BMS-936559 is a humanized high affinity anti-PD-L1 monoclonal antibody that blocks PD-L1 binding to PD-1 and CD80. Brahmer et al. (2012) reported a clinical trial regarding the activity of BMS-936559 in advanced cancer. A total of 200 patients were enrolled, 17 of whom were ovarian cancer patients. The results demonstrated that at a 10 mg/kg dose, 1 ovarian cancer patient achieved a partial response, and 3 ovarian cancer patients achieved stable disease. Avelumab, a human anti-PD-L1 antibody, could specifically bind to PD-L1 and block the links with PD-1 (Heery et al., 2017). Disis et al. (2019) described a cohort study on the effect of avelumab in refractory or relapsed ovarian cancer. They enrolled 125 patients and treated them with 10 mg/kg avelumab every 2 weeks. The results demonstrated that 12 patients had an objective response. Among them, 1 patient had a complete response. Additionally, the median PFS and OS were 2.6 and 11.2 months, respectively.

Immune checkpoints are an important part of maintaining self-tolerance. Immune checkpoint therapy enhancing antitumor immune responses via suppressing inhibitory pathways in T cells. Currently researched suggest that immune checkpoint inhibition is a potential way to activate the immune response. But further exploration is needed to improve the cure rate.

## Conclusion

**FIGURE 3:**
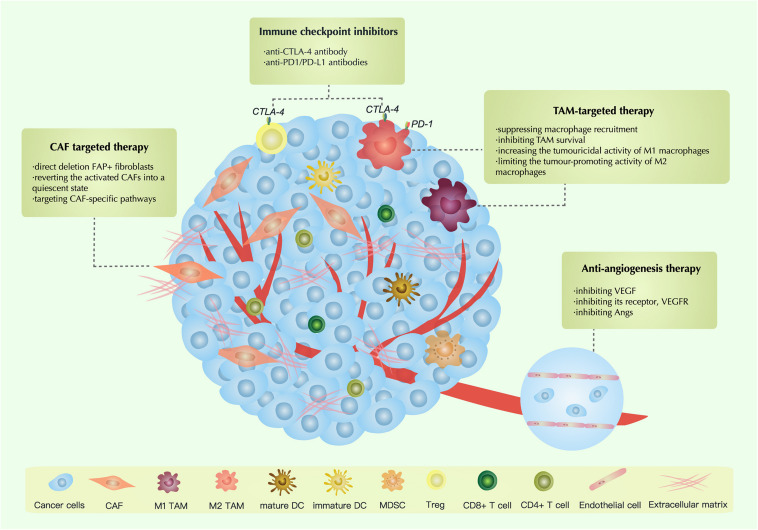
Tumor microenvironment related therapeutic strategies in ovarian cancer. The graph shows multiple strategies targeted the tumor microenvironment in ovarian cancer. Among them, several strategies are currently in clinical use, while others are at different phases of clinical development. CAFs: Cancer-associated fibroblasts; TGF-β: Transforming growth factor-β; TAMs: Tumor-associated macrophages; CCL2: chemokine (C-C motif) ligand 2; CSF-1R: colony stimulating factor-1 (CSF1) receptor; VEGF: Vascular endothelial growth factors; VEGFR: Vascular endothelial growth factor receptor.

The current standardized treatment for ovarian cancer is optimal cytoreductive surgery plus platinum-based chemotherapy with the carboplatin–paclitaxel regimen (Bolton et al., 2012). Nevertheless, due to chemotherapy-resistant and refractory diseases, the sensitivity of chemotherapy decreases, thereby decreasing the long-term survival rate and increasing the recurrence rate (Lim and Ledger, 2016). Currently, the TME is regarded as a possible therapeutic target for ovarian cancer. In this review, we summarized new targeted therapies at the interface between ovarian cancer and the TME (Figure 3): Several ways are adopted to target ovarian TME. CAFs targeting therapy includes direct deletion FAP+ fibroblasts, reverting the activated CAFs into a quiescent state, and targeting CAF-specific pathways; Anti-angiogenesis is an important TME targeting therapeutic strategy. Since VEGF is the most typical activator of angiogenesis, anti-angiogenesis therapy is divided into three types: anti-VEGF, inhibitors of VEGF receptors and Angs inhibitors. Among them, bevacizumab, the first FDA-approved anti-angiogenesis antibody, plays a crucial role in anti-angiogenesis therapy for ovarian cancer. TAM-targeted antitumor strategies also have drawn much research attention. Currently, the present TAM-targeted therapeutics consist of (i) suppressing macrophage recruitment (such as CCL2 inhibitors and CSF-1R inhibitors); (ii) inhibiting TAM survival (e.g., bisphosphonates); (iii) increasing the tumouricidal activity of M1 macrophages (for example, agonists of the NF-κB signaling pathway such as TLR-7 agonist and other agents such as IL-12); and (iv) limiting the tumor-promoting activity of M2 macrophages (inhibitor of STAT3). Finally, increasing evidence of the therapeutic effect of immune checkpoint inhibitors in ovarian cancer has been reported. Immune checkpoint inhibitors limit inhibitory pathways in T cells, thereby enhancing antitumor immune responses. In particular, CTLA-4 and PD1/PD-L1 are important immune checkpoints for ovarian cancer. We also summarized the ongoing clinical trials targeting ovarian cancer tumor microenvironment (Table 3). Although new therapeutic approaches targeting the TME could not cure ovarian cancer, they showed the potential to control the development of ovarian cancer. Besides, with the development of “-omic” technology, scientists are unveiling a more detailed “territory” of ovarian cancer TME; the data drawn from this area is certain to facilitate novel therapy exploration, with the expectation to bring breakthrough discoveries to this deadly disease. We believe that TEM-targeted strategies should be applied in ovarian cancer as a valuable adjuvant therapy.

**TABLE 3:** Ongoing clinical trials targeting ovarian cancer tumor microenvironment (TME).

Drug	Combination therapy	Simple size	Status	Trial number	Phase
**Target VEGF**					
Sevacizumab	Paclitaxel; Topotencan	48	Recruiting	NCT03763123	1
Bevacizumab	Paclitaxel; Ricolinostat	6	Terminated	NCT02661815	1
Bevacizumab	Carboplatin; Paclitaxel	9	Completed	NCT01219777	1
Bevacizumab	Niraparib	108	Active, not recruiting	NCT02354131	1,2
Bevacizumab	Carboplatin; Paclitaxel; Rucaparib	234	Recruiting	NCT03462212	1,2
Bevacizumab	–	27	Unknown	NCT02022917	2
Bevacizumab	–	35	Recruiting	NCT02884648	2
Bevacizumab	–	36	Completed	NCT00748657	2
Bevacizumab	–	40	Not yet recruiting	NCT03611179	2
Bevacizumab	–	64	Completed	NCT00022659	2
Bevacizumab	Gemcitabine; Carboplatin; Cisplatin; Oxaliplatin	7	Terminated	NCT01936974	2
Bevacizumab	Paclitaxel; Cisplatin	20	Completed	NCT00511992	2
Bevacizumab	Irinotecan	29	Completed	NCT01091259	2
Bevacizumab	Topotecan	40	Completed	NCT00343044	2
Bevacizumab	Gemcitabine; Carboplatin	45	Completed	NCT00267696	2
Bevacizumab	RAD001	50	Completed	NCT01031381	2
Bevacizumab	Tocotrienol	60	Recruiting	NCT04175470	2
Bevacizumab	Erlotinib; Paclitaxel; Carboplatin	60	Completed	NCT00520013	2
Bevacizumab	Paclitaxel; Carboplatin	62	Completed	NCT00129727	2
Bevacizumab	Anetumab Ravtansine; paclitaxel	96	Recruiting	NCT03587311	2
Bevacizumab	Niraparib	106	Active, not recruiting	NCT03326193	2
Bevacizumab	Fosbretabulin Tromethamine	107	Completed	NCT01305213	2
Bevacizumab	Everolimus	150	Unknown	NCT00886691	2
Bevacizumab	Paclitaxel; Carboplatin	190	Completed	NCT00937560	2
Bevacizumab	Temsirolimus	252	Completed	NCT01010126	2
Bevacizumab	Cyclophosphamide	20	Completed	NCT00856180	3
Bevacizumab	Capecitabine; Carboplatin; Oxaliplatin; Paclitaxel	50	Active, not recruiting	NCT01081262	3
Bevacizumab	Carboplatin; Paclitaxel	100	Active, not recruiting	NCT03635489	3
Bevacizumab	Rucaparib	190	Not yet recruiting	NCT04227522	3
Bevacizumab	Paclitaxel; Carboplatin; PLD; Gemcitabine	406	Unknown	NCT01802749	3
Bevacizumab	Carboplatin; Paclitaxel	1021	Completed	NCT01239732	3
Bevacizumab	Paclitaxel; Carboplatin	400	Unknown	NCT01706120	4
**Target VEGFR**					
Apatinib	Fluzoparib	98	Active, not recruiting	NCT03075462	1
Chiauranib	–	25	Completed	NCT03166891	1,2
BIBF 1120	–	32	Completed	NCT01669798	2
JI-101	–	31	Active, not recruiting	NCT01853644	2
Regorafenib	–	43	Recruiting	NCT02736305	2
Tivozanib	–	31	Active, not recruiting	NCT01853644	2
Apatinib	Albumin-bound paclitaxel	35	Not yet recruiting	NCT03942068	2
Apatinib	PLD	150	Recruiting	NCT04348032	2
Cediranib	Olaparib	4	Completed	NCT02340611	2
Pazopanib	Paclitaxel	118	Active, not recruiting	NCT02383251	2
Regorafenib	Tamoxifen	68	Active, not recruiting	NCT02584465	2
Cediranib	Laparib; Paclitaxel; Pegylated Liposomal Doxorubicin Hydrochloride; Topotecan	680	Recruiting	NCT02502266	2,3
Cediranib	Olaparib	618	Recruiting	NCT03278717	3
**Target CTLA-4**					
Tremelimumab	Olaparib	50	Recruiting	NCT02571725	1,2
Tremelimumab	Olaparib	170	Recruiting	NCT04034927	2
**Target PD-1**					
ABBV-181	SC-003	74	Terminated	NCT02539719	1
PDR001	Ribociclib; Fulvestrant	60	Recruiting	NCT03294694	1
Nivolumab	COM701	140	Recruiting	NCT03667716	1
Pembrolizumab	Modified Vaccinia Virus Ankara Vaccine Expressing p53	28	Recruiting	NCT03113487	1
Pembrolizumab	AMG386	60	Active, not recruiting	NCT03239145	1
Nivolumab	Varlilumab	175	Completed	NCT02335918	1,2
Pembrolizumab	Carboplatin	29	Active, not recruiting	NCT03029598	1,2
Pembrolizumab	PLX3397	78	Terminated	NCT02452424	1,2
Pembrolizumab	Galinpepimut-S	90	Recruiting	NCT03761914	1,2
Pembrolizumab	Niraparib	122	Active, not recruiting	NCT02657889	1,2
Pembrolizumab	ENB003	130	Not yet recruiting	NCT04205227	1,2
Sintilimab	Manganese Chloride; nab-paclitaxel; Platinum chemotherapy	80	Recruiting	NCT03989336	1,2
TSR042	Niraparib	150	Recruiting	NCT03955471	2
Nivolumab	Rucaparib	1	Active, not recruiting	NCT03824704	2
Pembrolizumab	–	100	Active, not recruiting	NCT02644369	2
Pembrolizumab	–	376	Active, not recruiting	NCT02674061	2
Pembrolizumab	Gemcitabine; Cisplatin	21	Active, not recruiting	NCT02608684	2
Pembrolizumab	Carboplatin	22	Not yet recruiting	NCT04387227	2
Pembrolizumab	Carboplatin; Paclitaxel	30	Recruiting	NCT02766582	2
Pembrolizumab	DPX-Survivac; Cyclophosphamide	42	Recruiting	NCT03029403	2
Pembrolizumab	DPX-Survivac; Cyclophosphamide	184	Recruiting	NCT03836352	2
SHR-1210	Famitinib	265	Recruiting	NCT03827837	2
Nivolumab	TSR-042; Chemotherapy Drugs	196	Not yet recruiting	NCT03651206	2,3
Nivolumab	Rucaparib	1012	Recruiting	NCT03522246	3
TSR-042	Niraparib	1228	Recruiting	NCT03602859	3
**Target PD-L1**					
Atezolizumab	Carbplatin, Cyclophophamide	12	Active, not recruiting	NCT02914470	1
Atezolizumab	RO6870810	36	Terminated	NCT03292172	1
Atezolizumab	Carboplatin; Paclitaxel; Niraparib; Gemcitabine; PLD	414	Recruiting	NCT03598270	1
MEDI4736	Eribulin	9	Active, not recruiting	NCT03430518	1
MEDI4736	Focal radiotherapy	22	Recruiting	NCT03283943	1
Atezolizumab	DEC-205/NY-ESO-1 Fusion Protein CDX-1401; Guadecitabine; Poly ICLC	75	Suspended	NCT03206047	1,2
Avelumab	Entinostat	140	Active, not recruiting	NCT02915523	1,2
MEDI4736	PLD; Motolimod;	53	Active, not recruiting	NCT02431559	1,2
MEDI4736	ONCOS-102	78	Recruiting	NCT02963831	1,2
TQB2450	Anlotinib	30	Not yet recruiting	NCT04236362	1,2
Atezolizumab	Vigil	25	Active, not recruiting	NCT03073525	2
Avelumab	–	5	Terminated	NCT03312114	2
MEDI4736	Azacitidine	28	Active, not recruiting	NCT02811497	2
MEDI4736	TPIV200	29	Active, not recruiting	NCT02764333	2
Atezolizumab	Carboplatin; Paclitaxel; Niraparib; Gemcitabine; PLD	414	Recruiting	NCT03598270	3
Avelumab	PLD	566	Active, not recruiting	NCT02580058	3
**Target VEGF combined with VEGFR**					
Bevacizumab	BIBF 1120	21	Completed	NCT02835833	1
Bevacizumab	Sorafenib	55	Completed	NCT00436215	2
**Target VEGF combined with PD-1**					
Bevacizumab	Pembrolizumab	40	Not yet recruiting	NCT03596281	1
Bevacizumab	TSR042; Niraparib	40	Active, not recruiting	NCT03574779	2
Bevacizumab	Pembrolizumab; Cyclophosphamide	40	Active, not recruiting	NCT02853318	2
Bevacizumab	Pembrolizumab; Olaparib	44	Not yet recruiting	NCT04361370	2
Bevacizumab	Pembrolizumab; Carboplatin; Paclitaxel	45	Not yet recruiting	NCT03275506	2
Bevacizumab	Nivolumab; Rucaparib	76	Recruiting	NCT02873962	2
Bevacizumab	Pembrolizumab; Carboplatin; Paclitaxel	1086	Recruiting	NCT03740165	3
Bevacizumab	TSR042; Niraparib; Carboplatin; Paclitaxel	337	Not yet recruiting	NCT03806049	3
**Target VEGF combined with PD-L1**					
Bevacizumab	MEDI4736; Olaparib	427	Active, not recruiting	NCT02734004	1,2
Bevacizumab	Atezolizumab; Carboplatin; Paclitaxel	40	Recruiting	NCT03394885	1,2
Bevacizumab	Avelumab; M6620; Carboplatin; Paclitaxel; Gemcitabine; PLD	3	Completed	NCT03704467	2
Bevacizumab	Atezolizumab; Cobimetinib	29	Recruiting	NCT03363867	2
Bevacizumab	MEDI4736; Olaparib	74	Active, not recruiting	NCT04015739	2
Bevacizumab	Atezolizumab; Acetylsalicylic acid	160	Recruiting	NCT02659384	2
Bevacizumab	Atezolizumab; Pegylated Liposomal Doxorubicin Hydrochloride	488	Suspended	NCT02839707	2,3
Bevacizumab	Avelumab; Chemotherapy	79	Active, not recruiting	NCT03642132	3
Bevacizumab	Atezolizumab; Platinum-based chemotherapy	614	Active, not recruiting	NCT02891824	3
Bevacizumab	Atezolizumab; Chemotherapy	664	Recruiting	NCT03353831	3
Bevacizumab	MEDI4736; Olaparib; Carboplatin; Paclitaxel	1056	Recruiting	NCT03737643	3
Bevacizumab	Atezolizumab; Paclitaxel; Carboplatin	1300	Active, not recruiting	NCT03038100	3
**Target VEGFR combined with PD-1**					
Apatinib	SHR-1210	28	Not yet recruiting	NCT04068974	1
Lenvatinib	Pembrolizumab	180	Active, not recruiting	NCT03797326	2
**Target Angs combined with PD-1**					
AMG386	Pembrolizumab	60	Active, not recruiting	NCT03239145	1
**Target Angs combined with VEGF**					
MEDI3617	Bevacizumab; Paclitaxel; Carboplatin	162	Completed	NCT01248949	1
**Target CTLA-4 combined with PD-1**					
Ipilimumab	Nivolumab	48	Recruiting	NCT03508570	1
Ipilimumab	Nivolumab	5	Terminated	NCT03342417	2
Ipilimumab	Nivolumab	62	Recruiting	NCT03355976	2
Tremelimumab	Nivolumab	100	Active, not recruiting	NCT02498600	2
**Target CTLA-4 combined with PD-L1**					
Tremelimumab	MEDI4736; Chemotherapy	61	Recruiting	NCT03249142	1,2
Tremelimumab	Olaparib; MEDI4736	36	Recruiting	NCT02953457	2
Tremelimumab	MEDI4736	100	Recruiting	NCT03026062	2

*PLD, pegylated liposomal doxorubicin.*

## Author Contributions

YFY and JY drafted the manuscript. YY designed all figures and made substantial revision to the original manuscript. XZ and XW checked and modified the manuscript. All authors contributed to the article and approved the submitted version.

## Conflict of Interest

The authors declare that the research was conducted in the absence of any commercial or financial relationships that could be construed as a potential conflict of interest.
